# The spatial organization of sphingofungin biosynthesis in *Aspergillus fumigatus* and its cross-interaction with sphingolipid metabolism

**DOI:** 10.1128/mbio.00195-24

**Published:** 2024-02-21

**Authors:** Katarina Jojić, Fabio Gherlone, Zoltán Cseresnyés, Alexander U. Bissell, Sandra Hoefgen, Stefan Hoffmann, Ying Huang, Slavica Janevska, Marc Thilo Figge, Vito Valiante

**Affiliations:** 1 Biobricks of Microbial Natural Product Syntheses, Leibniz Institute for Natural Product Research and Infection Biology - Hans Knöll Institute (Leibniz-HKI), Jena, Germany; 2 Faculty of Biological Sciences, Friedrich Schiller University Jena, Jena, Germany; 3 Applied Systems Biology, Leibniz Institute for Natural Product Research and Infection Biology - Hans Knöll Institute (Leibniz-HKI), Jena, Germany; 4 (Epi-)Genetic Regulation of Fungal Virulence, Leibniz Institute for Natural Product Research and Infection Biology - Hans Knöll Institute (Leibniz-HKI), Jena, Germany; Stony Brook University, Stony Brook, New York, USA

**Keywords:** sphingofungins, sphingolipid, *Aspergillus fumigatus*, secondary metabolites, cellular compartments

## Abstract

Sphingofungins are sphinganine analog mycotoxins acting as inhibitors of serine palmitoyl transferases, enzymes responsible for the first step in the sphingolipid biosynthesis. Eukaryotic cells are highly organized with various structures and organelles to facilitate cellular processes and chemical reactions, including the ones occurring as part of the secondary metabolism. We studied how sphingofungin biosynthesis is compartmentalized in the human-pathogenic fungus *Aspergillus fumigatus*, and we observed that it takes place in the endoplasmic reticulum (ER), ER-derived vesicles, and the cytosol. This implies that sphingofungin and sphingolipid biosynthesis colocalize to some extent. Automated analysis of confocal microscopy images confirmed the colocalization of the fluorescent proteins. Moreover, we demonstrated that the cluster-associated aminotransferase (SphA) and 3-ketoreductase (SphF) play a bifunctional role, supporting sphingolipid biosynthesis, and thereby antagonizing the toxic effects caused by sphingofungin production.

**IMPORTANCE:**

A balanced sphingolipid homeostasis is critical for the proper functioning of eukaryotic cells. To this end, sphingolipid inhibitors have therapeutic potential against diseases related to the deregulation of sphingolipid balance. In addition, some of them have significant antifungal activity, suggesting that sphingolipid inhibitors-producing fungi have evolved mechanisms to escape self-poisoning. Here, we propose a novel self-defense mechanism, with cluster-associated genes coding for enzymes that play a dual role, being involved in both sphingofungin and sphingolipid production.

## INTRODUCTION

Fungi are known as a source of several secondary metabolites (SMs) (1). In fungi, SMs are normally synthetized by enzymes, whose co-regulated genes are neighboring each other in the genome, forming so-called biosynthetic gene clusters (BGCs). So far, BGCs have been studied mainly at the genomic level, focusing on their evolution and activation, and at the genetic level, elucidating the role of gene products, enzymes, in multi-step catalysis. Furthermore, recent studies on the spatial organization of secondary metabolism have contributed to a better understanding of the chemical logic of SMs in fungi. The knowledge gained can potentially be used to further implement research into drug discovery, development, and production, as reported for some enzymes involved in the biosynthesis of β-lactams (2, 3), trichothecene (4, 5), fumonisins (6), aflatoxin (7, 8)*,* viriditoxin (9), mycophenolic acid (10), melanin (11, 12), and perylene quinones (13), which have been found in distinct cellular compartments.

**Fig 1:**
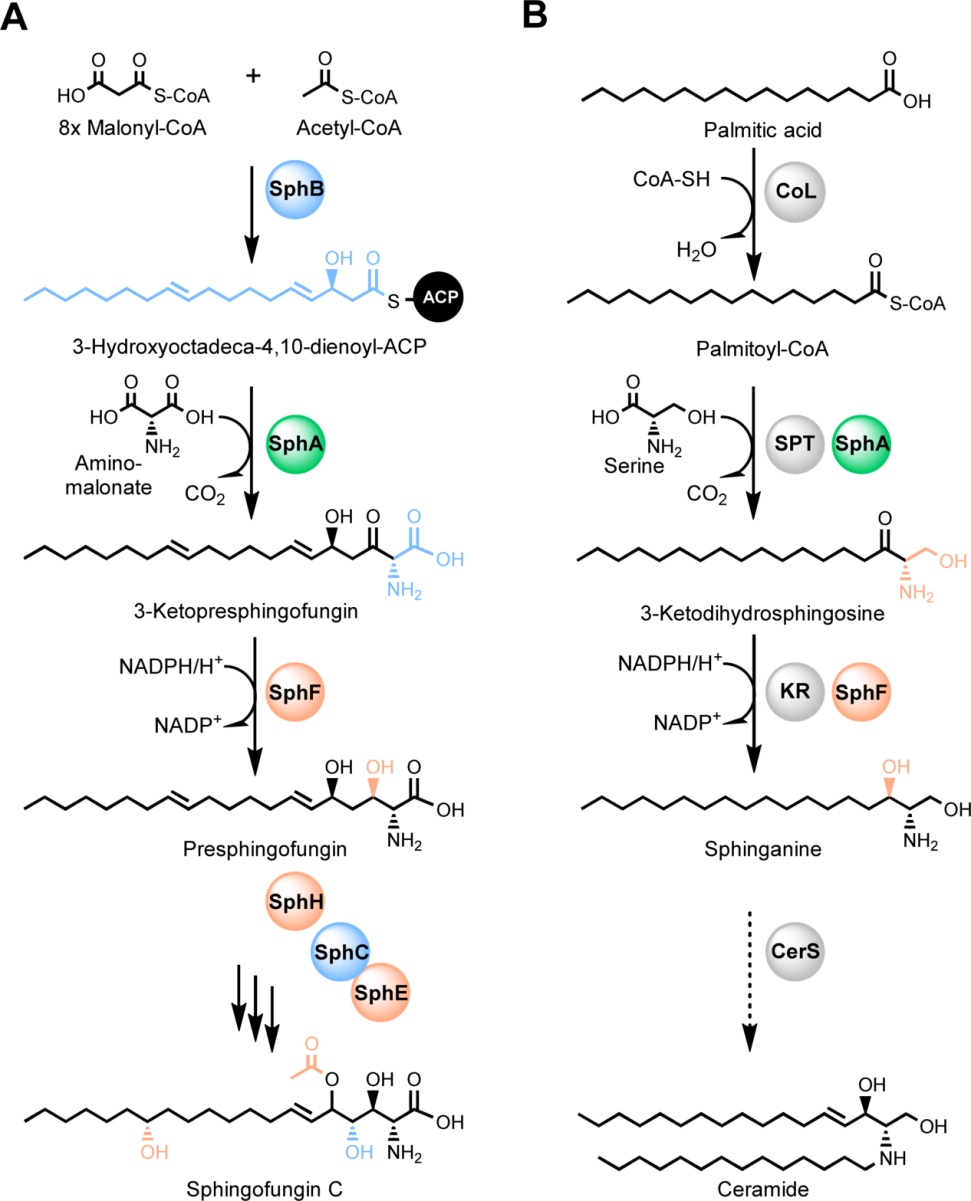
Cross-talk interaction between the sphingolipid and sphingofungin biosynthetic pathways in *Aspergillus fumigatus*. (**A**) Schematic representation of the sphingofungin pathway and localization of the involved biosynthetic enzymes—cytosolic (blue), vesicular compartments (orange), and both locations (green). (**B**) Schematic representation of the biosynthesis of ceramide, the relevant precursor of sphingolipids. SphB, polyketide synthase; SphA, aminotransferase; SphF, 3-ketoreductase; SphH, cytochrome P450 monooxygenase; SphC, monooxygenase; SphE, acyl transferase; CoL, acyl-CoA ligase; SPT, serine palmitoyl transferase; KR, 3-ketoreductase; CerS, ceramide synthase.

Sphingofungins are a family of polyketid-derived compounds first isolated from *Aspergillus fumigatus* and then later reported in *Paecilomyces variotii* and *Aspergillus penicillioides* (14–16). These compounds are toxic metabolites that inhibit serine palmitoyl transferase (SPT), a multimeric enzyme responsible for the condensation of palmitoyl-CoA with serine, the first and crucial step in the biosynthesis of sphingolipids (SLs) (17). Recently, we succeeded in elucidating the biosynthesis of sphingofungin B, C, and D in *A. fumigatus* (18) and assayed their potential to inhibit SPTs (19). In brief, the polyketide synthase (PKS) SphB together with the aminotransferase SphA generate the first intermediate. Additional modification of the backbone by SphF (3-ketoreductase), SphH (P450 monooxygenase), SphE (acetyl transferase), and SphC (monooxygenase) yields the final product sphingofungin C (Fig. 1A; Fig. S1).

SLs are essential structural components of the eukaryotic cell membrane. In addition to their structural function, they also have other important roles in cell signaling and communication, differentiation, and apoptosis (20). The dysregulation of the sphingolipid metabolism in humans can result in various diseases, including cancer, diabetes, and neurodegenerative diseases like Alzheimer’s disease (21). As such a vital cellular process, the SL biosynthesis is highly regulated. In eukaryotes, *de novo* synthesis of sphingolipids takes place in the ER and the first biosynthetic step, the condensation of L-serine with palmitoyl-CoA leading to the formation of 3-ketosphinganine, is catalyzed by the SPT (20–22). The functionality of the SPT has been well characterized in humans. It is a membrane-bound heterodimeric protein complex composed of SPTLC1/SPTLC2 as the core subunits, which is connected to the ER membrane (23–25). In yeast, the two subunits are named Lcb1 and Lcb2 (26). Notably, this is different in sphingolipid-producing bacteria, where the SPT is a soluble homo-dimeric protein (27). Later steps in SL biosynthesis involve the formation of ceramide and phytoceramide, which are the hub precursor molecules for the synthesis of more complex sphingolipids (Fig. 1B) (21).

In this work, we performed localization studies of the enzymes responsible for the SL and sphingofungin biosynthesis in *A. fumigatus*. Confocal microscopy revealed that the two pathways are partially co-compartmentalized in the ER and ER-derived vesicles. By employing computational image analysis, we evaluated the colocalization and obtained information on how the fluorescent proteins behave within the cell. The obtained results prompted the question of how the toxin and its target occupy the same compartments, and which potential mechanisms of self-resistance are involved. To this end, our *in vivo* and *in vitro* experiments revealed that two enzymes associated with the sphingofungin biosynthesis, the aminotransferase (SphA) and 3-ketoreductase (SphF), can function as part of SL biosynthesis and thus help the fungus to reduce self-poisoning effects (Fig. 1). Moreover, we elucidated in more detail the terminal domains responsible for the localization in the ER and ER-derived vesicles.

## RESULTS

### Enzymes involved in sphingofungin biosynthesis exhibit distinct cellular localization

Our first aim was to identify a protein that could be used to specifically label the ER and the ER-derived vesicle in *A. fumigatus*. Since in *Aspergillus nidulans*, the gene *lcbA* was confirmed to code for a functional SPT, similar to *LCB1* from *S. cerevisiae* (28), we used the deduced amino acidic sequence as a bait and identified its homolog in *A. fumigatus* (AFUB_078390). We could exploit two *S*. *cerevisiae* thermosensitive mutant strains, which host gene variants of *LCB1* and *LCB2*, named as *lcb1-2* and *lcb2-2*, respectively (29), to validate the functionality of the identified gene. The complementation of the *lcb1-2* mutant in yeast with the isolated cDNA, confirmed that LcbA from *A. fumigatus* can build a functional SPT complex with Lcb2 from *S. cerevisiae* (Fig. S2).

**Fig 2:**
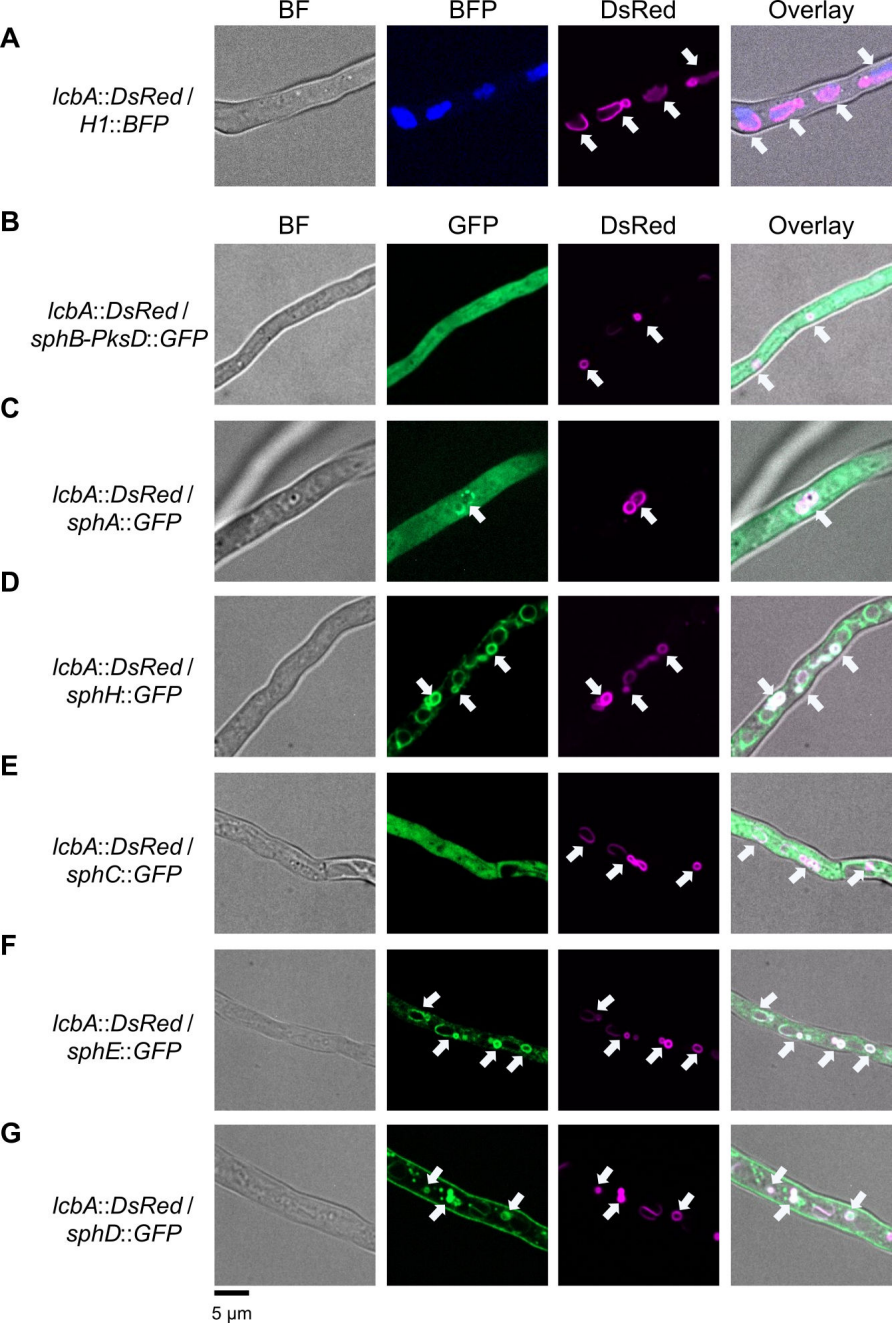
Confocal microscopy for the localization analysis of sphingofungin biosynthetic enzymes. (**A**) The LcbA-DsRed fusion enzyme was used to label the endoplasmic reticulum (ER) and the ER-derived vesicles, while the tagged Histone1-BFP was used to label nuclei. The colocalization studies were performed by co-expression of genes coding for the LcbA-DsRed with (**B**) SphB-PksD-GFP, (**C**) SphA-GFP, (**D**) SphH-GFP, (**E**) SphC-GFP, (**F**) SphE-GFP, and (**G**) SphD-GFP. Shown are individual channels—GFP, BFP, and DsRed magenta and overlay with bright-field (BF) images. Arrows indicate ER-derived vesicles.

LcbA from *A. fumigatus* was then tagged with a red fluorescent protein (LcbA-DsRed) and co-expressed with a histone H1 tagged with a blue fluorescent protein (BFP), for nuclear localization (30), confirming that it is assigned to ER and ER-derived vesicles (Fig. 2A). Control strains were checked for both DsRed and GFP fluorescence in order to exclude any bleed-through phenomenon (Fig. S3).

The BGC responsible for sphingofungin production was already identified in *A. fumigatus* (18). In order to determine the subcellular localization of the enzymes involved, each of them was tagged with a C-terminal GFP. We started with the backbone-forming enzyme, the PKS SphB. Since the tagging of the complete protein gave no detectable signals, we decided to tag a smaller portion, the acyl transferase domain (PksD), with a C-terminal GFP. Microscopy revealed that this chimeric protein, the SphB-PksD-GFP, is mainly accumulated in the cytosol (Fig. 2B).

Since SphB has no recognizable thioesterase domain and is located in the cytosol, we expected the aminotransferase SphA, which “collects” the polyketide and uses it as a substrate, to colocalize with the PKS. In fact, SphA is mainly detected in the cytosol, but occasionally co-compartmentalizes with LcbA, revealing a possible dual localization (Fig. 2C). Next, the C-terminal tagging of the ketoreductase SphF, the enzyme involved in the second step of biosynthesis, produced only weak signals, and its localization could not be determined with certainty (Fig. S4). Nonetheless, the analysis of the P450 monooxygenase SphH showed that even though the gene product is mainly allocated in the vesicles, it is also clearly present in the perinuclear ER (Fig. 2D). Time-lapse microscopy performed with the tagged *sphH*-expressing strain revealed that these ER-derived vesicles move through the hyphae, toward the growing tip and, in some cases, they come into contact with the plasma membrane (Movie S1).

The next investigated enzyme was SphC, the monooxygenase responsible for the formation of the C-4 hydroxyl group, which was found in the cytosol (Fig. 2E). Interestingly, SphE, which is responsible for the acetylation and formation of the end product sphingofungin C, was present in the ER and ER-derived vesicles (Fig. 2F). Finally, SphI, encoded by a cluster-associated gene, to which previously we could not assign a function (18), was localized to the cytosol as well (Fig. S5). These results indicate that the biosynthesis of sphingofungins in *A. fumigatus* occurs in both the cytosol and ER-derived vesicles.

As with many other BGCs identified in fungi, the sphingofungin cluster contains an MFS (Major Facilitator Superfamily) transporter. Tagging it with GFP revealed that it is localized to several compartments, namely the plasma membrane, the ER, ER-derived vesicles, punctuate, as well as mobile vesicles (<1 µm) (Fig. 2G; Movie S2).

Finally, since peroxisomes are also known to host biosynthetic enzymes of distinct secondary metabolites (8, 31), in order to exclude the involvement of this additional compartment, we created strains that expressed GFP-tagged Pex3, a peroxisomal membrane protein, and DsRed-tagged LcbA or SphH. Microscopy confirmed that the locations of these proteins are distinct from each other (Fig. S6).

### Enzyme colocalization revealed by quantitative image analysis

**Fig 3:**
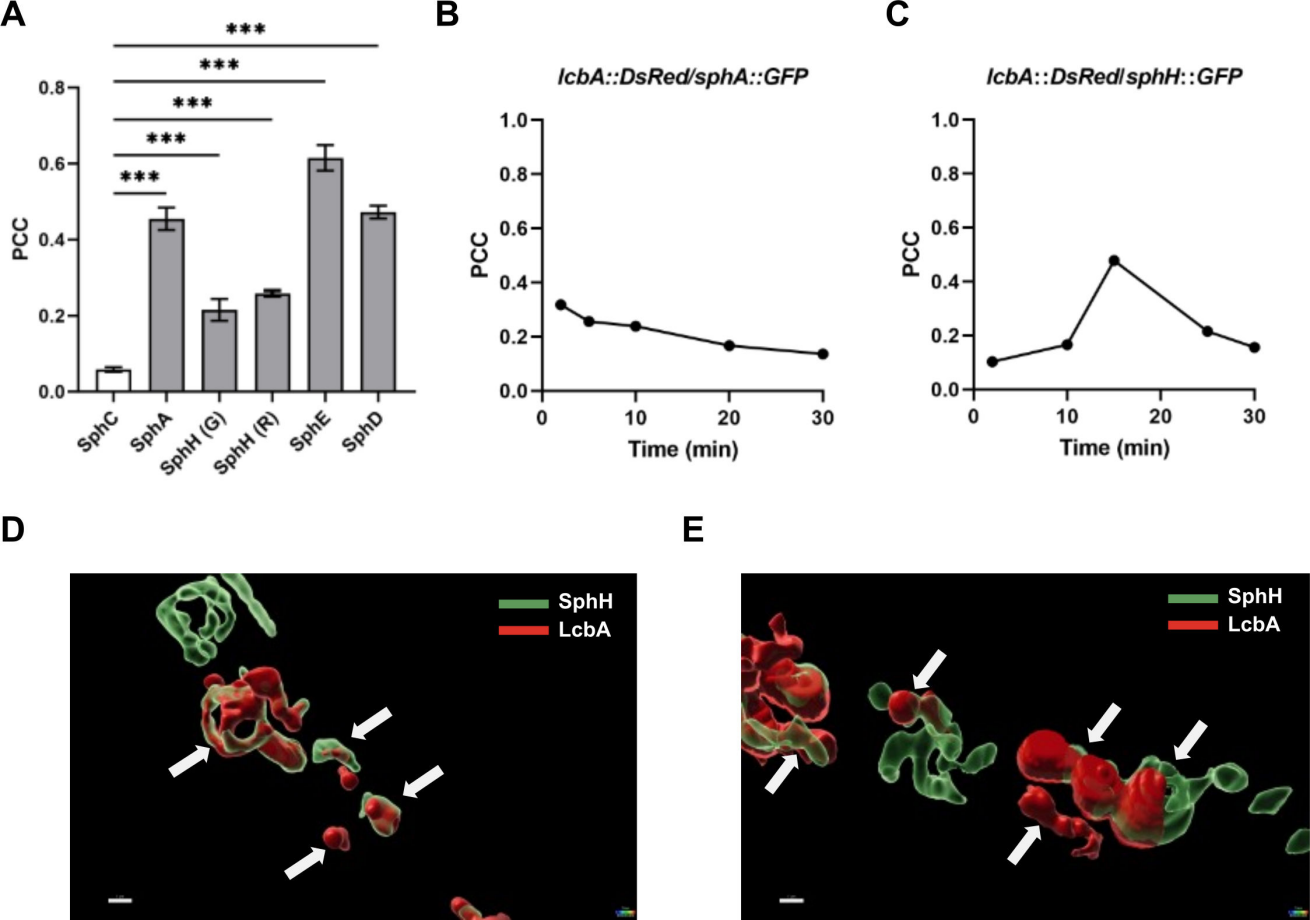
Quantification of colocalization of LcbA with selected sphingofungin enzymes: (**A**) Values obtained from 3D images of fixed fungal hyphae. The data columns show mean values with ±SEM (number of data points = 19–37) of two independent biological samples. The Pearson’s correlation coefficient (PCC) was determined based on the overlapping pixels (red and green) in the analyzed images. The cytosolic enzyme SphC, was used as negative control. For SphH, the exchange of the fluorescent tag, green (G) or red (R), gave a similar PCC value. One-way ANOVA followed by Dunnett’s multiple comparisons test was used to compare (***P < 0.001). (**B**) Changes of PCC values in live cells for (B) SphA and (**C**) SphH suggest a decrease of the red signal over the time. (**D and E**) 4D imaging obtained using snapshots of different regions of the hyphae of SphH (G) strain. As shown, LcbA and SphH are located in close proximity but their fluorescent signals do not always overlap, indicating the presence of micro-regions on endosomes and explaining the low PCC value measured in (A). Indicated scale bar is 1 µm. Arrows indicate overlapping objects in ER-derived vesicles.

The microscopy experiments gave us information about the cellular localization of the enzymes involved in sphingofungin biosynthesis. Here, we observed a difference in the intensity distribution of LcbA-DsRed and the GFP-tagged enzymes. We developed automated image processing algorithms to analyze 3D images of fixed fungal cells to evaluate and quantify the degree of colocalization in the various strains. The computational analysis revealed whether the distribution of the signal intensities is linearly correlated between two channels (Pearson’s correlation coefficient, PCC) (32). The strain expressing the tagged cytosolic enzyme SphC was used as a control. As expected, the correlation between SphC-GFP and LcbA-DsRed was very low, although not zero. It is possible that a very bright cytosolic fluorescence signal bleeds into the signal of the compartmentalized LcbA, producing artifacts, to a small extent. On the contrary, the results obtained for compartmentalized sphingofungin enzymes exhibited a positive correlation (PCC > 0.5), which confirms what was observed experimentally (Fig. 3A). However, the tagged SphH showed the lowest correlation (PCC ~ 0.2), which was not in agreement with what we observed by looking at the microscopy pictures, where the SphH-GFP was always associated to the ER and ER-derived vesicles. We then decided to make additional strains expressing this time LcbA-GFP and SphH-DsRed, but the exchange of fluorophores had no impact on the calculated values (Fig. 3A).

To study this phenomenon in more detail, we performed an additional 4D microscopy in live cells, calculating the PCC at various time points. We assayed two strains—one encoding SphA-GFP and LcbA-DsRed, while the second SphH-GFP and LcbA-DsRed. In the case of SphA, in a live cell, the correlation was steadily decreasing with time (Fig. 3B). Even so, its presence in the vesicles was confirmed (Fig. S7; Movie S3). For SphH, the correlation was changing more sharply, with both lower and higher values (Fig. 3C). Additional 4D object analysis showed that, although SphH and LcbA are in the same compartment, the two proteins are not overlapping at all times (Movie S4). Masking of the green channel over the red made it possible to appreciate the various changes of the organelles, showing that SphH (green) and LcbA (red) are associated with different sub-domains of the ER, even forming separate microvesicles over time (Fig. 3D and E; Movie S5), explaining the lack of overlapping pixels and the low PCC values. These different subpopulations of vesicles have already been observed in eukaryotes, suggesting distinct functions during vesicle specialization, such as cargo transport and secretion (33). Nonetheless, the association of SphH to the ER and ER-derived vesicle was confirmed.

### Cross-interaction between sphingofungin and SL biosynthesis

**Fig 4:**
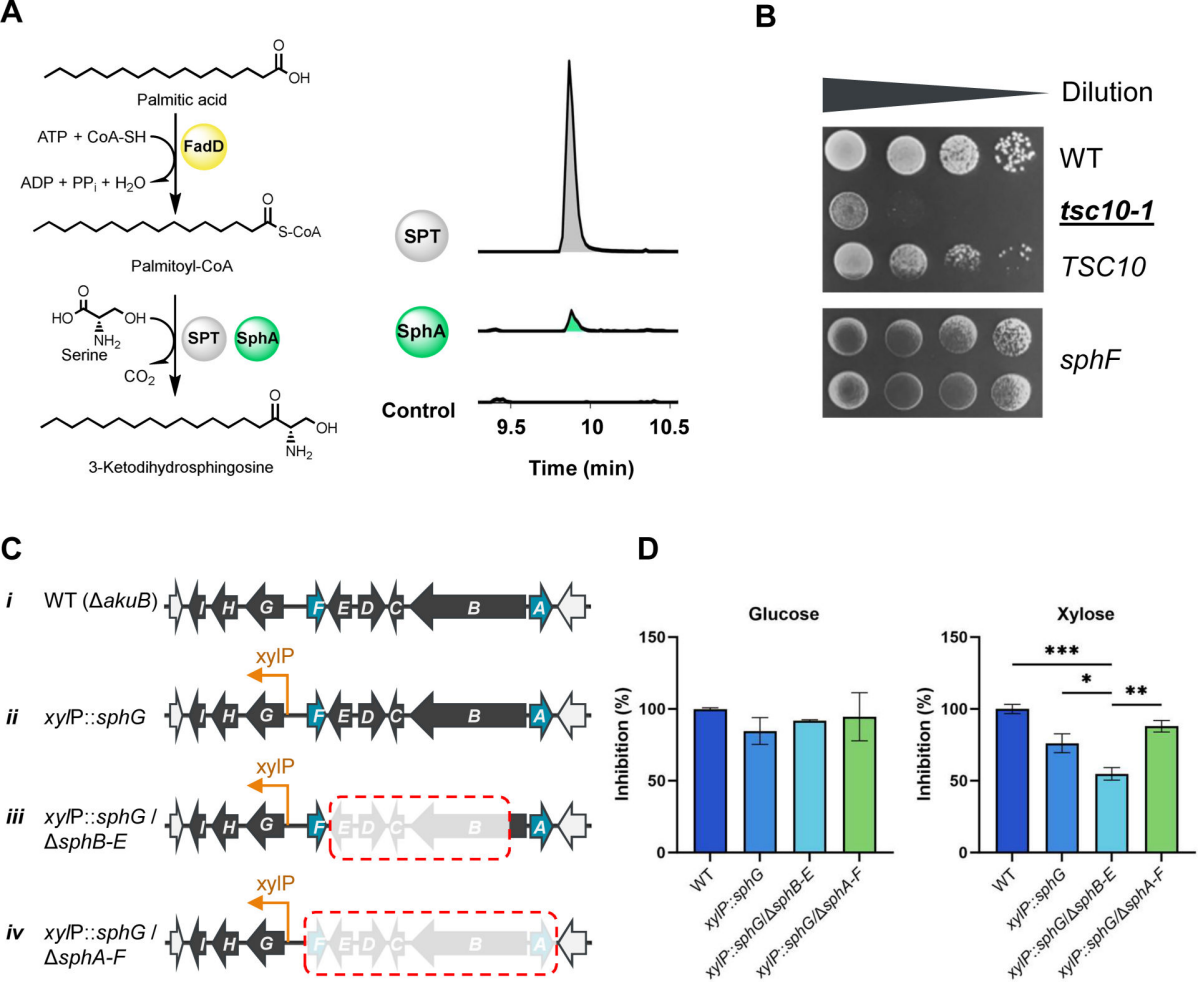
Analysis of SphA and SphF roles as an SPT and 3-ketoreductase in the sphingolipid biosynthesis. (**A**) *In vitro* assay assigning the function of SphA as an SPT. The product, 3-ketodihdrosphingosine, was analyzed with HPLC-HRMS. Shown are EIC overlays corresponding to *+m/z* 300.2897. Positive control was performed using SPT from *S. paucimobilis*. The palmitoyl-CoA was supplied by incubating palmitic acid, fee CoA and the long-fatty-acid CoA ligase, FadD, from *E. coli*. (**B**) The phenotype of the thermosensitive *S. cerevisiae tsc10-1*, unable to produce sphinganine, was recovered by expressing *sphF* from *A. fumigatus*. Plates were incubated at 35°C. (**C**) Schematic representation of the strain used for the sensitivity assay. (i) wild-type; (ii) strain over-expressing the *sphG* gene, coding for the cluster-associated transcription factor, under the control of a xylose inducible promoter (*xyl*P); (iii) strain over-expressing *sphA* and *sphF* but unable to produce sphingofungin; and (iv) strain lacking the sphingofungin biosynthetic pathway. (**D**) Resazurin assay performed with the obtained *A. fumigatus* strains in presence of 50 µM myriocin. Data shown are mean values ± SEM (*n* = 3). For statistical analysis, one-way ANOVA followed by Dunnett’s multiple comparisons test was used (*P < 0.05, **P < 0.01, and ***P < 0.001).

The localization of the aminotransferase SphA in both the cytosol and vesicles raised the question of whether this enzyme could have a dual function. Since SphA catalyzes the condensation of the non-proteinogenic amino acid aminomalonate with a polyketide during sphingofungin biosynthesis, we investigated if this enzyme can act as an SPT by supplying additional 3-ketodihydrosphingosine. We purified the enzyme directly from *A. fumigatus* and established an *in vitro* assay. To ensure the required palmitoyl-CoA for the reaction, we supplied palmitic acid to the reaction mix and additionally incubated the purified *Escherichia coli*-derived fatty-acid-CoA ligase FadD, together with SphA (34). Although the production of 3-ketodihydrosphinganine was lower compared to the SPT positive control [isolated from *Sphingomonas paucimobilis* (19)], we confirmed that SphA has SPT activity and that its dual functionality is driven by the availability of the used substrates (Fig. 4A).

Another gene that attracted our attention was SphF. This ketoreductase is responsible for the reduction of the C-3 keto group present in 3-ketopresphingofungin. Because of the similarity between 3-ketopresphingofungin and 3-ketodihydrosphinganine, we investigated whether SphF could also cross-interact with the sphingolipid biosynthesis. It is already been proven that the ketoreductase from the fumonisin cluster, Fum13, is able to complement the *S. cerevisiae tsc10-1* mutant strain, a conditional mutant, which is deficient in 3-ketosphinganine reductase activity when grown at 35°C (35, 36). As shown, the expression of *sphF* completely restored growth at 35°C, confirming the dual role of this enzyme (Fig. 4B).

In order to better define the activity of these proteins *in vivo*, and their potential role in self-defense, we tested if the over-expression of *sphA* and *sphF* would impact the sensitivity of *A. fumigatus* against SPT inhibitors by employing the resazurin cell viability assay (37, 38). As background, we used the strain having the cluster-associated transcription factor *sphG* under the control of the strong inducible promoter (*xyl*P) (Fig. 4Ci-ii) (18). We created a strain where parts of the *sph* gene cluster were deleted, and a second strain with a larger portion deleted, including the *sphA* and *sphF* genes (Fig. 4Ciii-iv). In both cases, sphingofungin production was suppressed. The strains were tested against myriocin, a commercially available sphinganine-like compound, acting against the SPT (Fig. 4D; Fig. S8) (39). The results undoubtedly showed that during inducing conditions, with the presence of xylose in the media as carbon source and the lack of production of internal sphingofungin, the over-expression of *sphA* and *sphF* significantly reduced the toxicity of myriocin, confirming their role in aiding the sphingolipid production. Analysis of the gene expression with quantitative real-time PCR (qRT-PCR) of the aforementioned strains revealed high levels of expression of genes in the deletion mutants, including *sphA* and *sphF* (Fig. S9).

### SphH and SphF contain atypical helix-loop-helix terminal domains

After observing that the various enzymes involved in sphingofungin biosynthesis are fully or partially compartmentalized in the ER and in the ER-derived vesicles, we assumed to be able to locate possible signal peptide (SP) in their amino acid sequences. Surprisingly, all herein available prediction tools failed to detect the presence of potential SPs (Table S1). We thus proceeded to analyze the protein sequences via the Conserved Domain Database (CDD) (40), and examined portions, which were not part of the predicted active domains. We selected suitable N-terminal peptides for SphD, SphE, SphF, and SphH, which could function as potential SPs (Fig. S10), and fused them to GFP under the control of the *Tet*^ON^ promoter. *Aspergillus niger* was employed as the recipient strain for our initial experiments. The analyzed mutants did not show a clear localization pattern, with the only exception of the 66 amino acids long N-terminal peptide isolated from SphH.

**Fig 5:**
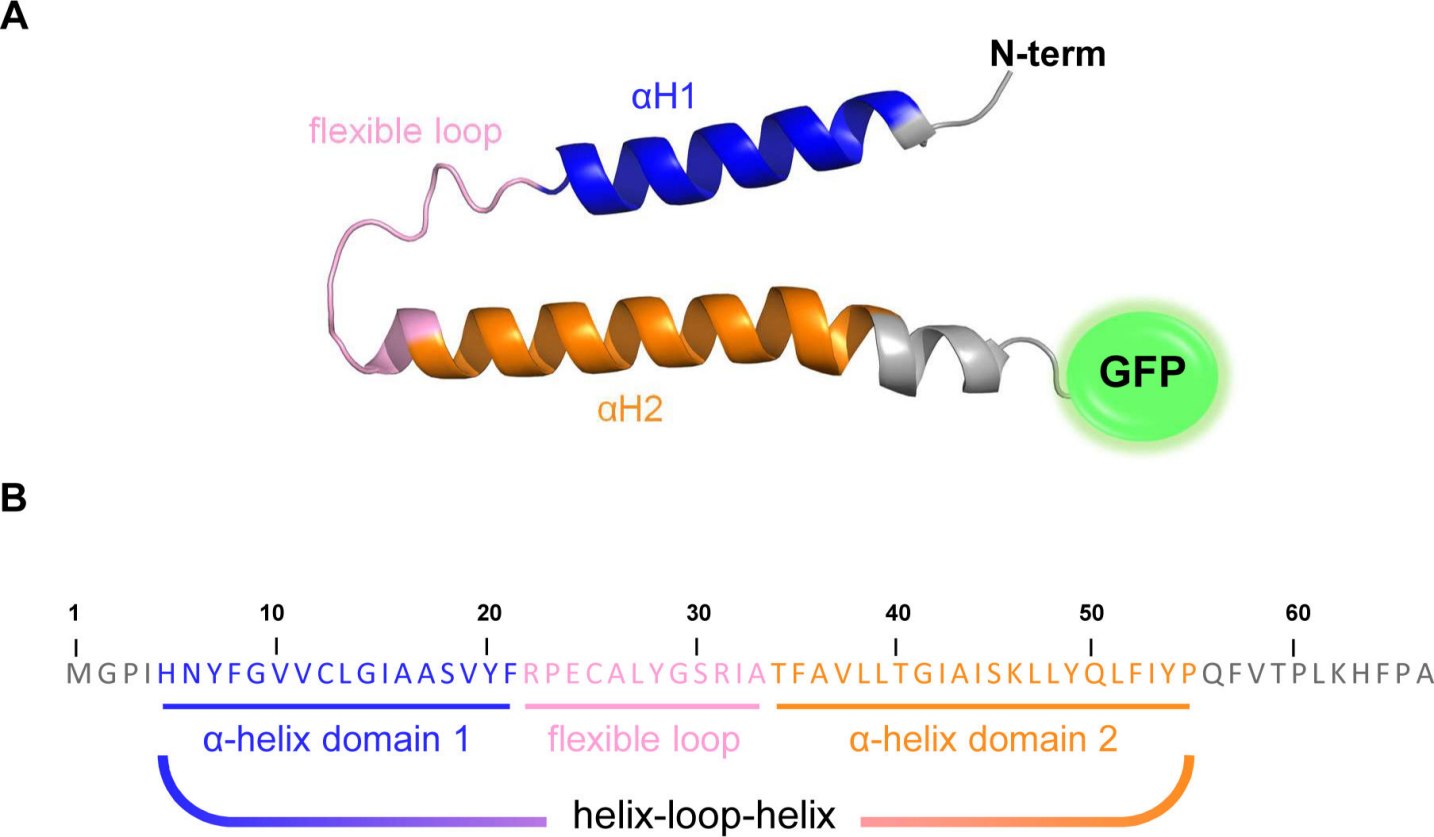
Predicted tertiary structure and functional domains of HLH^SphH^. (**A**) HLH^SphH^ was predicted to entail a first α-helix (αH1, blue), a flexible loop (pink), followed by a second α-helix (αH2, orange). The tertiary structure was predicted using AlphaFold2. (**B**) Sequence of HLH^SphH^ with its predicted membrane topology helix-loop-helix.

In order to better elucidate the structure of SphH (1–66), we submitted its amino acid sequence to AlphaFold2 prediction (41), revealing that the peptide contains two alpha helixes (αH1 and αH2) spaced by a flexible loop (Fig. 5). This was quite surprising, because N-terminal helix-loop-helix (HLH) structures have been mainly found in DNA-binding proteins, such as transcriptional regulators (42).

**Fig 6:**
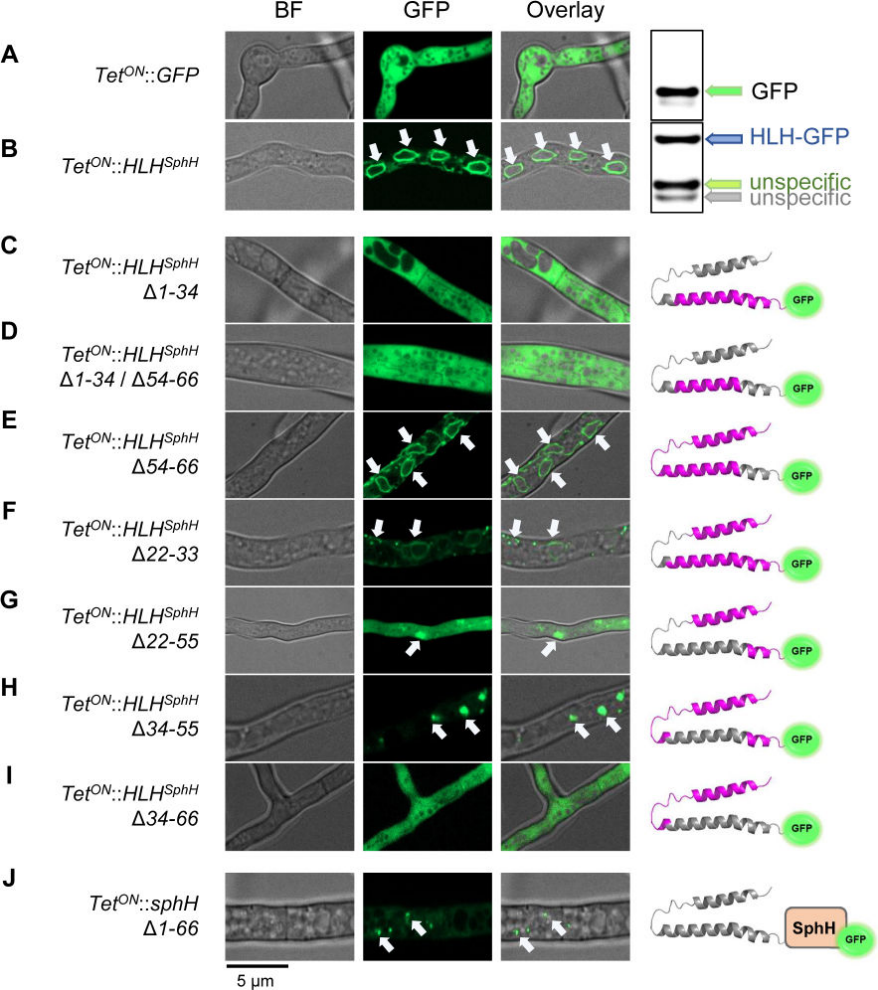
Confocal microscopy for the localization analysis of HLH^SphH^ mutants in *A. fumigatus*. Each construct is expressed under an inducible Tet^ON^ promoter and C-terminally tagged to a GFP fluorescent reporter. (**A**) GFP accumulated in the cytosol, with corresponding western blot detection (right). (**B**) GFP N-terminally tagged with HLH^SphH^, localized in the ER, with corresponding western blot detection. (**C–I**) Localization of HLH^SphH^ mutants with topology for each of the strains reported on the right side. The 3D structure of HLH^SphH^ is depicted highlighting the deleted amino acids in gray, and the ones still present in magenta. (**J**) The GFP-tagged SphH protein lacking its N-terminal HLH domain was unstable and mainly degraded. BF, bright-field. Arrows indicate ER-derived vesicles.

We then further characterized the topology of this domain and investigated which parts are essential to achieve correct cellular localization. We cloned the sequence coding for the first 66 amino acids upstream of *GFP*, confirming that the peptide can specifically target proteins to the ER (Fig. 6A and B). Moreover, western blot analysis showed that the fusion construct, HLH^SphH^-GFP was correctly translated, even if some unspecific degradation products were present (Fig. 6A and B, right).

We first removed the initial 34 amino acids of HLH^SphH^ (Δ1–34), which resulted in the removal of the αH1 and the flexible loop, observing that without these elements, the compartmentalization does not occur (Fig. 6C). Then, we removed the last 13 amino acids (Δ54–66) leaving the sole αH2 subdomain, and also in this case the GFP signal was in the cytosol (Fig. 6D). To confirm this, we created a variant with a Δ54–66 deletion, and indeed we could verify that this part of the HLH domain is not relevant for its functionality (Fig. 6E). Next, we analyzed the importance of the flexible loop, by removing it alone (Δ22–33) and in combination with the αH2, leaving the sole αH1 (Δ22–55). In both cases, we noticed low fluorescent signal, with mislocalized and aggregated fluoresces (Fig. 6F and G). Finally, the removal of αH2 domain (Δ34–55 and Δ34–66) gave back a mislocalized cytosolic fluorescence signal (Fig. 6H and I). The results obtained draw a clear picture of the investigated N-terminus domain, revealing that the HLH structure is important for the correct compartmentalization of the protein. Interestingly, the expression of a GFP-tagged SphH construct without the N-terminus HLH domain, resulted in the lack of a fluorescence signal, possibly due to misfolding of the chimeric protein (Fig. 6J).

In addition to *Aspergilli*, we could observe that the HLH^SphH^ was also functional in yeast, which gave the opportunity to investigate the functional parts of the peptide in *S. cerevisiae*. The results obtained in yeast largely confirmed what was observed in *A. fumigatus*, with the exception that in *S. cerevisiae*, the presence of the αH2 domain is sufficient for ER localization (Fig. S11). At the moment, we cannot explain this phenotype, which needs further investigation. Nonetheless, the inter-specie functionality of the N-terminus HLH domain was fully demonstrated.

**Fig 7:**
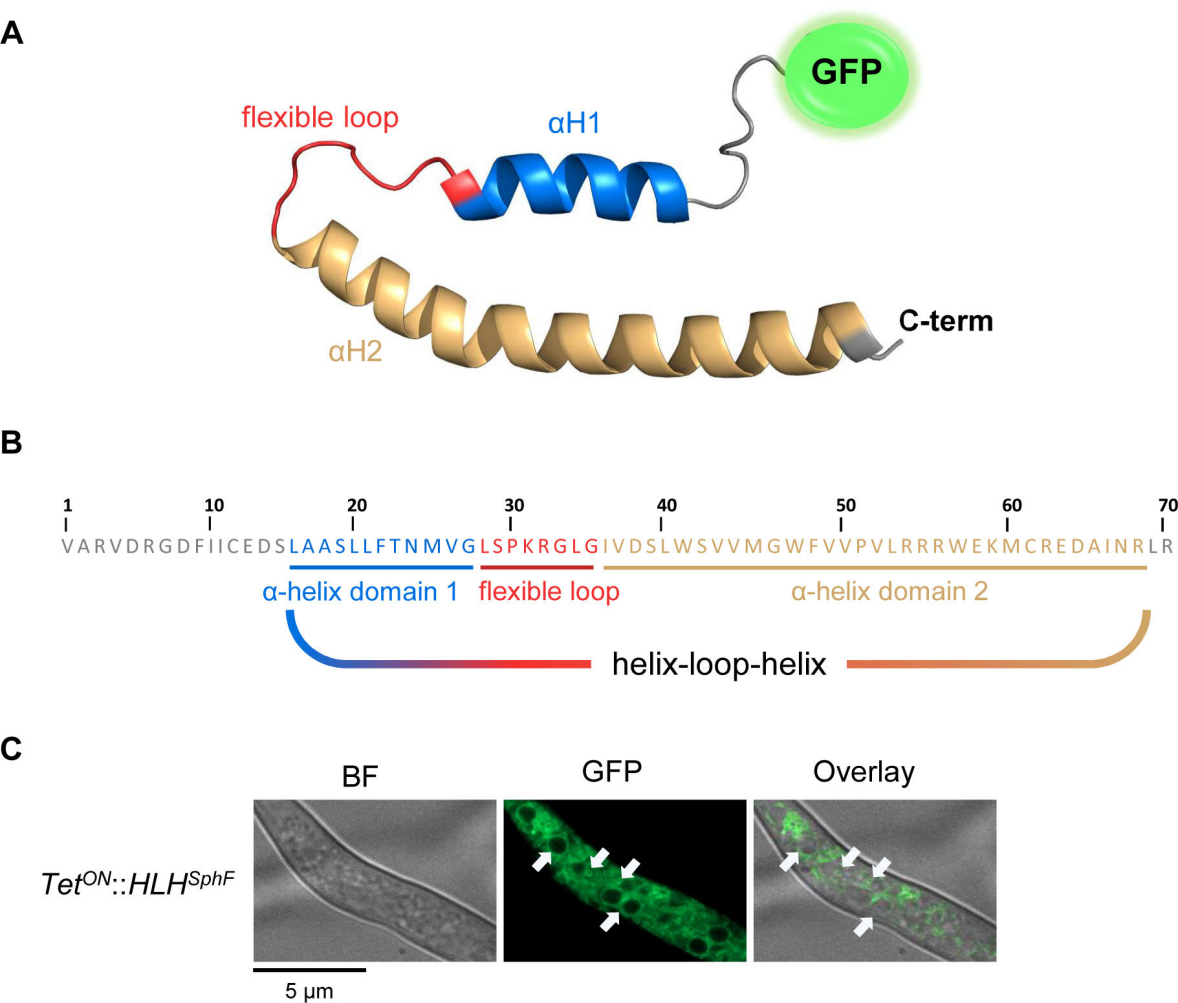
Localization analysis, predicted tertiary structure, and functional domains of HLH^SphF^. (**A**) HLH^SphF^ was predicted to entail a first α-helix (αH1, blue), a flexible loop (red), followed by a second α-helix (αH2, orange). The tertiary structure was predicted using AlphaFold2. (**B**) Sequence of HLH^SphF^ with its predicted membrane topology helix-loop-helix. (**C**) Confocal microscopy for the localization analysis of HLH^SphF^ in *A. fumigatus*. The construct is expressed under an inducible Tet^ON^ promoter and N-terminally tagged with GFP. Arrows indicate ER-derived vesicles.

Inspired by the obtained data, we decided to process all proteins involved in sphingofungin biosynthesis that showed ER association with AlphaFold2. We could not identify any commonly defined structure, but an HLH domain was found at the C-terminus of SphF (*HLH^SphF^*) (Fig. 7A and B). Based on the structure prediction, *GFP* was N-terminally fused to *HLH^SphF^*, and transferred to *A. fumigatus*. In this case, microscopy revealed the presence of small vesicles that developed upon induction, confirming the functionality of the identified C-terminal HLH (Fig. 7C).

### Alteration of SphC localization affects sphingofungin production

**Fig 8:**
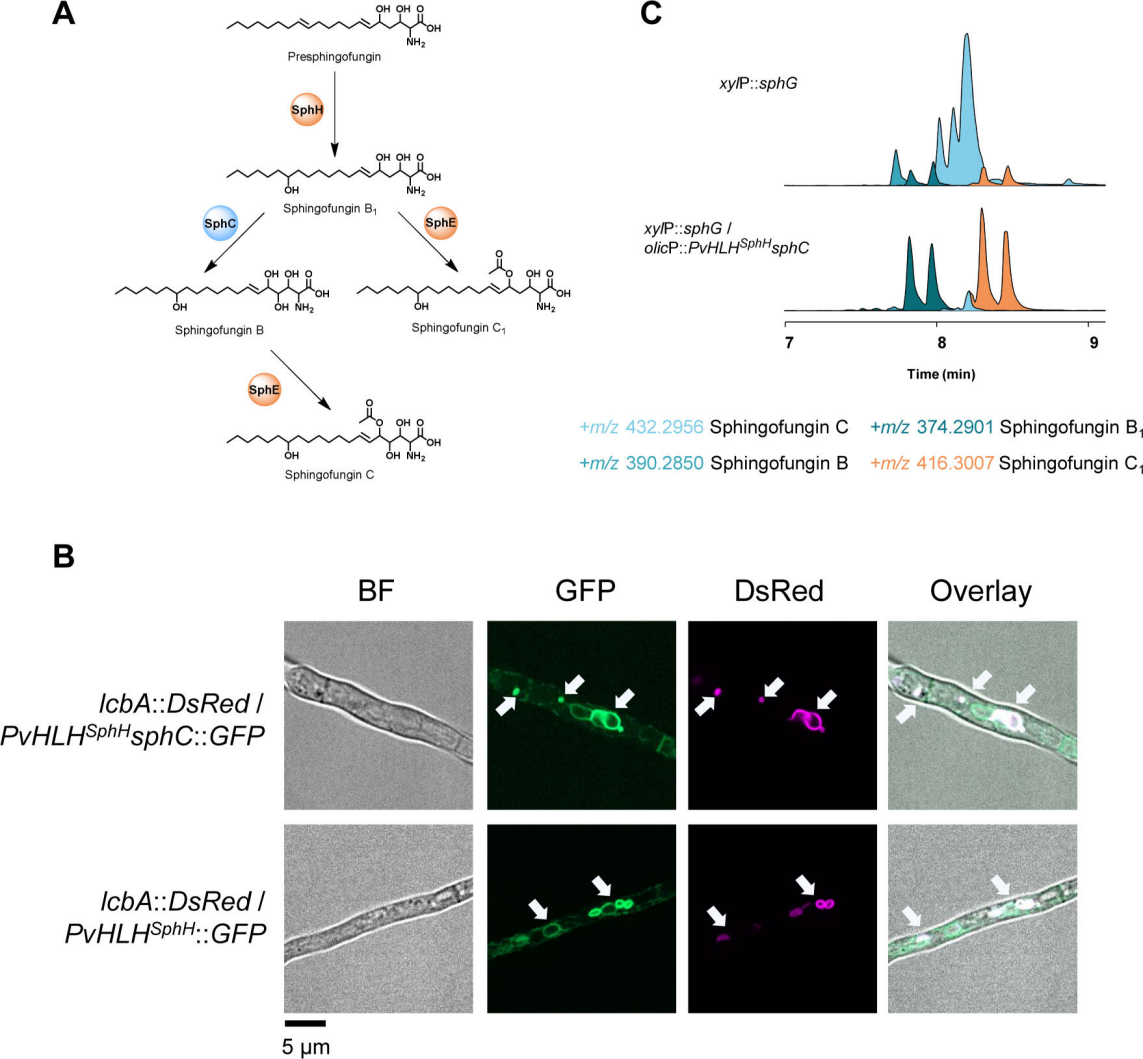
Production of sphingofungin intermediates upon ER-tagging of SphC. (**A**) The last stages of sphingofungin C production take place in the ER (enzymes in orange) and cytosol (SphC in blue). (**B**) Localization of the SphC enzyme fused with the PvHLH^SphH^ peptide at the N-terminus and GFP at C-terminus (upper panels) and of the PvHLH^SphH^ peptide directly tagged to the GFP (lower panels). Fluorescent signals were observed in parallel with the LcbA::DsRed for localization. (**C**) Constitutive activation of *sph* BGC leads to the accumulation of the end-product sphingofungin C, while changes in the localization of SphC, from the cytosol to the ER, strongly decrease its functionality, implementing the accumulation of shunt products. Shown are EIC overlays corresponding to *+m/z*. Arrows indicate ER-derived vesicles.

The analyses conducted so far have shown that in the last three steps of sphingofungin biosynthesis, there is a repeated translocation between ER and cytosol, with sphingofungin B_1_ produced by SphH in the ER, which is then modified by the cytosolic SphC (Fig. 8A). The last step is then performed again in the ER, with SphE that acetylates sphingofungin B, obtaining sphingofungin C. We decided to investigate if the translocation of SphC to the ER, and the close proximity to precursor-forming enzymes, would implement sphingofungin C production. In order to avoid unspecific recombination in the sphingofungin BGC, we used the HLH^SphH^ isolated from *P. variotii* and fused it to SphC N-terminus (Fig. 8B). The *HLH^SphH^-sphC* fusion construct was introduced in the *xylP::sphG* mutant under the control of the constitutive *olic*P promoter (18, 43). To our surprise, we detected very low amounts of the final product, sphingofungin C, and an increase of the shunt product sphingofungin C_1_ (Fig. 8C). This experiment revealed that the N-terminus tagging and/or change in SphC localization, negatively affects its activity.

## DISCUSSION

The compartmentalization of enzymes in eukaryotic cells is a crucial step for the proper functioning of many metabolic pathways. Although often considered separate from the primary metabolism, secondary metabolism utilizes common metabolic precursors. Thus, specific localization enables different metabolic activities to be performed simultaneously, establishing a spatial control of the various multi-step reactions occurring within the cell.

Our previous work on the compartmentalization of enzymes involved in the fumonisin biosynthesis revealed that two cluster-associated ceramide synthases, Fum17 and Fum18, are localized in the ER-derived vesicles, cohabiting with the sphingolipid biosynthesis (6). In that case, we were able to easily explain this phenomenon: the two enzymes increase the available ceramide and phytoceramide thereby reducing the toxic effects derived upon fumonisin production; thus, their localization in the ER is consequential to their function.

As sphingofungin biosynthesis is partially colocalized with the sphingolipid biosynthesis, it raised the question of possible self-resistance mechanisms. As previously reported, in fungi there are different mechanisms used to prevent self-poisoning upon the production of toxic compounds. These include the presence of detoxifying enzymes, as reported for gliotoxin and berkeleylactone E (44, 45), whereas for other mycotoxins, e.g., deoxynivalenol, MFS transporters contribute to secretion of the final product (46). As mentioned above for fumonisin, the BGC includes enzymes that provide further ceramide synthase activity (6). Here, we report a novel mechanism, with enzymes SphA and SphF having a dual role and actively interacting with the affected biosynthesis. In our previous work, we determined *in vitro* that when SphA is incubated with 3-hydroxyoctadec-4-enoyl-CoA, an esterified precursor similar to the SphB product, the condensation reaction took place only in the presence of aminomalonate, but not in the presence of serine (18). Here, we showed that SphA reproduces SPT activity by using serine as a substrate in the presence of palmitoyl-CoA. This suggests that the presence of SphA in proximity of ER-derived vesicles may aid sphingolipid biosynthesis. *In vivo* experiments performed with myriocin showed that the presence of SphA and SphF had a positive effect on the viability of the fungus, strongly implicating their role in decreasing self-poisoning effects, revealing an unprecedented mechanism. Furthermore, along with the *in vitro* data, we identified a novel homodimeric SPT (SphA) in eukaryotes.

The here presented localization studies confirmed that the first step in sphingofungin biosynthesis, the condensation of the polyketide with aminomalonate, occurs in the cytosol (18). Downstream, a total of three biosynthetic steps occur in the ER with SphF, SphH, and SphE as responsible enzymes. However, we found it rather peculiar that the second to last step of biosynthesis takes place in the cytosol and the last one returns to the ER but the change in SphC compartmentalization supports this catalytic hierarchy.

Time-lapse experiments revealed that SphH- and SphD-associated vesicles show a transient connection to the plasma membrane. However, it could not be determined with certainty whether some of the vesicles fuse with the cell membrane. Although the deletion of the *sphD* coding gene did not affect the growth of the mutant (18), its presence at different cellular sites, including the plasma membrane, suggests that the encoded MFS transporter is involved in the intra- and extracellular transport/export of the produced sphingofungins. On the other hand, we observed that SphH was always compartmentalized, but not present on the plasma membrane. Additionally, the 4D image analysis revealed that during vesicle specialization, this enzyme tends to cluster into microregions, which are gradually separated from the SPT complex. During hyphal growth, organelles harboring either SphH or LcbA could also be observed, suggesting a physical separation of the two enzymes upon division and specialization of the ER.

Finally, in starting this study, we could not make any *in silico* predictions regarding the putative cellular localization of the enzymes involved in sphingofungin biosynthesis. We observed that even the most advanced prediction softwares were unable to correctly analyze these sequences. Furthermore, our attempts to isolate any SPs also failed. Nevertheless, our experiments permitted the discovery of a novel domain, an HLH domain placed at the N-terminus, which is relevant for the correct localization of the SphH protein to ER membranes. This also showed that new prediction software, such as AlphaFold, can be instrumental in the discovery of structure-related functions. Indeed, a second HLH, located at the C-terminus of SphF, was also identified in this way, even though the peptides have very low amino acid similarity (<50%).

**Fig 9:**
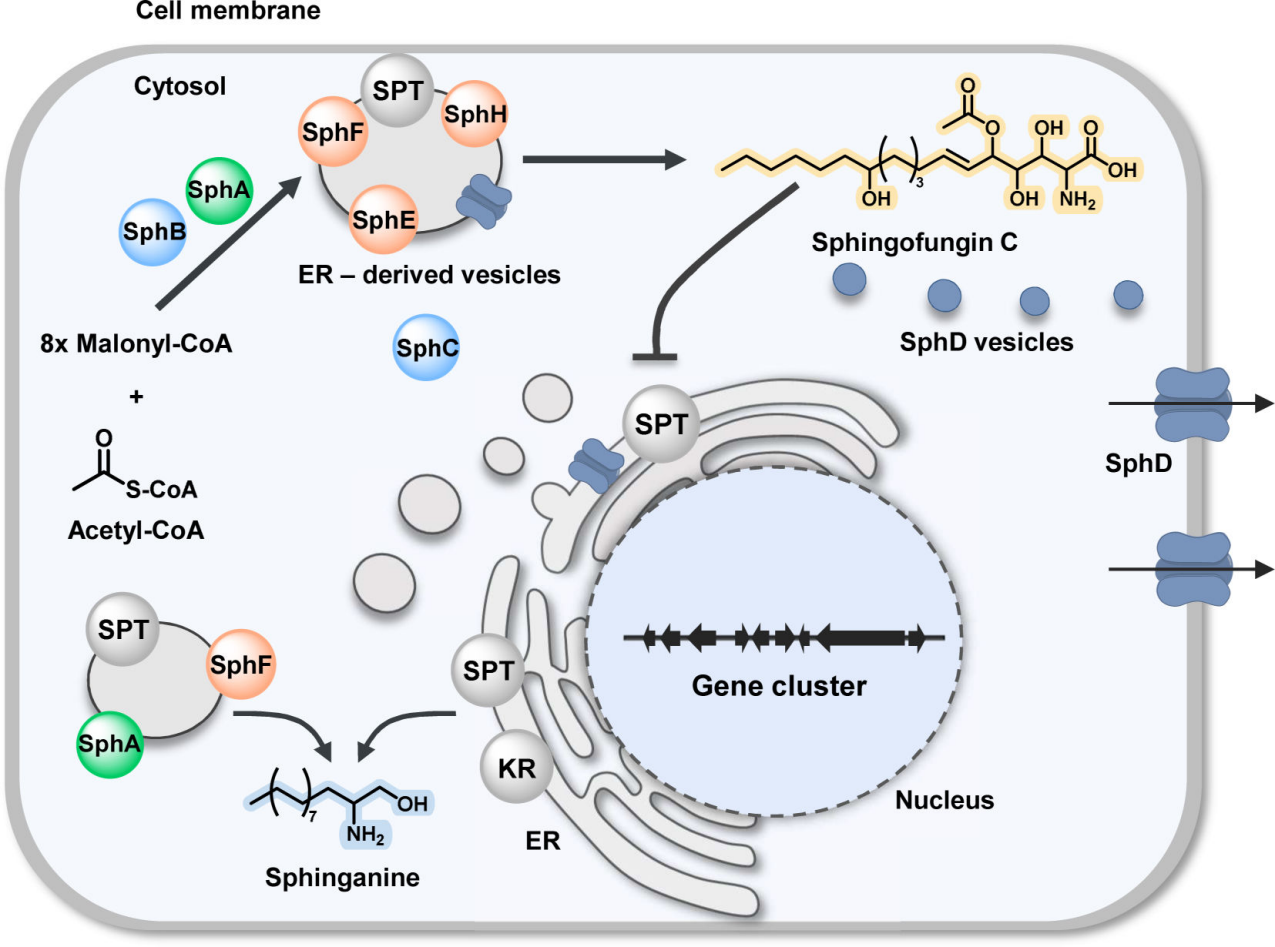
Compartmentalization of the sphingofungin biosynthetic cluster and proposed self-resistance mechanisms. The biosynthesis of sphingofungin starts in the cytosol, with the formation of the polyketide and the condensation with aminomalonate performed by the polyketide synthase SphB and the aminotransferase SphA. Further steps follow in the ER-derived vesicles. SphC, the enzyme responsible for the C-4 hydroxylation to give sphingofungin B and C, is in the cytosol. Toxic effects due by the self-inhibition of the SPT enzyme are reduced by the production of additional sphinganine from SphA and SphF. SphD, the MFS transporter, was found not only in the ER and ER-derived vesicles, but also widely distributed in the cell membrane.

In summary, we isolated two terminal domains responsible for ER-localization. We also analyzed the functionality of the novel HLH domain in detail, providing information on the structure and subdomains relevant for the correct compartmentalization to the ER and ER-derived vesicles in fungi. Additionally, we fully elucidated the localization of the sphingofungin biosynthetic enzymes, with steps occurring in the ER, ER-derived vesicles and the cytosol (Fig. 9). Finally, we propose that SphA and SphF play a dual role, as part of sphingolipid biosynthesis, revealing an additional role for BGC-associated genes hitherto overlooked.

## MATERIALS AND METHODS

### Strains and cultural conditions

*A. fumigatus*, *A. niger*, and *S. cerevisiae* strains that were used in this study are listed in Table S2 and S3. *A. fumigatus* strains were grown at 37°C on *Aspergillus* minimal media (AMM). *A. fumigatus* CEA17 Δ*akuB* strain (47) was grown on media supplemented with uracil and uridine with the final concentration of 20 µg/mL. When necessary, the media were supplemented with pyrithiamine (Sigma-Aldrich, Merck, Darmstadt, Germany) or hygromycin B (InvivoGen Europe, Toulouse, France) with the final concentration of 0.1 µg/mL and 200 µg/mL, respectively. *S. cerevisiae* strains were grown on YPD or selective SD at 30°C.

### Plasmid construction

All plasmids that were used in this study are listed in Table S4. Plasmid assembly was achieved using either transformation-associated recombination (TAR) cloning (48) or the seamless cloning method (49). Genes contained in the plasmids were amplified using Phusion High Fidelity DNA Polymerase (Fisher Scientific, Schwerte, Germany). Oligonucleotide primers that were used are listed in Table S5. The following genes were amplified using the primers listed: *sphA* [1 + 2], ORF for the PksD of *sphB* [3 + 4], *sphC* [5 + 6], *sphD* [7 + 8], *sphE* [9 + 10], *sphF* [11 + 12, 13 + 14], *sphH* [15 + 16], *sphI* [17 + 18], *lcbA* [19 + 20], and *pex3* 21 + 22]. Each of them was cloned into *Nco*I*-*digested pNDH-OGG (50) to receive GFP overexpression plasmids. To construct the plasmid that contained DsRed and the pyrithiamine cassette, first, the *Swa*I restriction site was introduced using primers [23 + 24], then, the pyrithiamine cassette was amplified from the pYES2-ptrA-Tet (18) vector using the [25 + 26] primer pair. Finally, all fragments were assembled into the *Pme*I/*Nco*I-digested pNDH-ODT vector (50). The obtained plasmid was subsequently digested with *Swa*I for the construction of the DsRed overexpression plasmids with PCR products amplified using primers *lcbA* [27 + 28] and *sphH* [29 + 30]. For tagging of histone H1, the gene was amplified with primers [31 + 32]. BFP sequence was obtained with [33 + 34] primers from EBFP2-N1 (Addgene #54595). Both parts were cloned into the *Not*I/*Nco*I*-*digested pNDH-OGG.

In order to obtain the backbone plasmid for the tagging of the N-terminal signal peptide candidates, pYES-PyrG-TetON (18) was used and amplified with primers [35 + 36]. GFP was amplified from pNDH-OGG (50) with primers [37 + 38] and both parts were assembled into the plasmid pYES-PyrG-TetON-GFP. The obtained plasmid was digested with *SfoI* and assembled with the amplified N-terminal sequences: *SphD (*1–77) [39 + 40], *SphE (*1–76) [41 + 42], *SphF (*1–52) [43 + 44], and *HLH^SphH^* [45 + 46].

The backbone for tagging of the C-terminal *HLH^SphF^* was obtained by linearizing the plasmid pYES2-PyrG-Tet^ON^ (18) with primers [105 + 35]. The sequence of *HLH^SphF^* was amplified from the genomic DNA of *A. fumigatus* using the primers [106 + 107], and fused to GFP, which was amplified from plasmid pYES2-pyrG-Tet^ON^-GFP. Final plasmid assembly was achieved using the seamless cloning method (44).

For the SPT complementation vectors, following genes were amplified with primer pairs: *S. cerevisiae LCB1* [47 + 48], *LCB2* [49 + 50], and *A. fumigatus lcbA* [51 + 52]. For the complementation of the 3-ketoreductase mutant *tsc10-1*, *TSC10* was amplified using [53 + 54] and *sphF* with [55 + 56] primers. Each of the genes was cloned into the *Bam*HI/*Sma*I-digested pYES2-TEF vector (51).

In order to achieve the expression of tagged proteins for ER and nuclear localization in *S. cerevisiae* (Fig. S10), the recombinant genes coding for the fluorescent proteins were expressed as a dicistron in combination with a selected auxotrophic marker, and inserted in the locus were the marker gene was previously deleted, namely Δ*met15*, Δ*leu2*, *and* Δ*his3. MET15* flanking regions were amplified using primer combinations [57 + 58] and [59 + 60], along with *2A::MET15* [61 + 62], *LAG1::DsRed* with [63 + 64]. The fragments were then fused in the *Hin*dIII/*Xba*I-digested pYES2 vector (Life Technologies, Darmstadt, Germany). *HIS3* flanking regions were amplified from *S. cerevisiae* prototrophic strain STI25221 (Jena Microbial Research Collection, Jena, Germany) gDNA with primers [65 + 66] and [67 + 68], while the constitutive *Tef* promoter was amplified with primers [69 + 70] from pYES2-TEF, and NLS from the pV2A-T vector (51) with [69 + 70], the *2A::LEU2* cassette was amplified with primers [61 + 73] and finally, *BFP* sequence from the EBFP2-N1 plasmid with [74 + 75]. The fragments were cloned into the *Hin*dIII/*Xba*I-digested pYES2 vector and then re-amplified and used for transformation.

In order to generate the mutant library of HLH^SphH^, site-directed mutagenesis was performed on the pYES2-TEF vector harboring the WT *HLH^SphH^* coding sequence with *GFP*. For each mutagenesis, the whole plasmid was reamplified. The following truncations were generated using primers stated here: *HLH^SphH^* Δ1–34 [76 + 77], *HLH^SphH^* Δ54–66 [78 + 79], *HLH^SphH^* Δ22–33 [80 + 81], *HLH^SphH^* Δ22–55 [81 + 82], *HLH^SphH^* Δ34–55 [82 + 83], *HLH^SphH^* Δ34–66 [83 + 84]. The truncation *HLH^SphH^* Δ1–34/Δ54–66, was obtained by site-directed mutagenesis on the plasmid *pTEF-HLH^SphH^* Δ54–66 using primers [76 + 77].

Plasmid encoding SP fused to SphC was assembled in the following way: flanking regions were amplified from *A. fumigatus* WT gDNA using primer pairs [85 + 86] and [87 + 88]. For the translocation, HLH^SphH^ from *P. varioti* was used and the fragment was amplified using primers [89 + 90] (18). Constitutive promoter *olic*P was amplified from the pNDH-OGG plasmid (50) with [91 + 92]. Finally, the pyrithiamine resistance cassette was amplified from pYES2-ptrA-Tet (18) using primer pair [93 + 94]. All the fragments were assembled in *Hin*dIII/*Xba*I-digested vector. For the localization study, PvHLH^SphH^ with sphC was amplified from the corresponding mutant, along with only the PvHLH^SphH^ sequence using primers pairs [95 + 96] and [95 + 97]. Both were cloned into the *Nco*I-digested pNDH-OGG vector.

### Fungal transformation and verification of mutants

To achieve the expression of the tagged genes, circular plasmids were transformed into the *A. fumigatus* CEA10 (52) and *A. niger* AB4.1 (53) strains according to the standard protocol. Mutants for the expression of tagged *lcbA* with *DsRed* and *GFP* were constructed, and the positive mutants were selected with 100 µg/mL of pyrithiamine or 200 µg/mL of hygromycin. Furthermore, the obtained single mutants were transformed with plasmids containing the tagged genes of the sphingofungin cluster or the gene coding for the histone H1 protein with the appropriate resistance cassette. Positive transformants were selected on plates containing both markers. The same principle was used to achieve the expression of the tagged *pex3* (AFUB_053830) for the visualization of peroxisomes. In order to check the mutants for the ectopic integration, primers were used that include the tagged genes with GFP [98 + 99] and DsRed [98 + 100] (Fig. S13). For *A. niger*, strains were selected on AMM plates and screened for GFP fluorescence on the confocal microscope.

For analysis of the *HLH^SphH^* mutant library in *A. fumigatus*, the peptide sequences were C-terminally fused to GFP and assembled *in vivo* using an AMA1-based vector (54). First, the N-terminal sequences were amplified from the above-mentioned pTEF plasmids using the primers [101 + 102], while GFP was amplified from the plasmid pYES2-pyrG-Tet^ON^-GFP with primers. The sequence *pyrG::Tet^ON^* was also amplified from the plasmid pYES2-pyrG-Tet^ON^-GFP with primers [104 + 35]. *A. fumigatus pyrG^-^* Δ*akuB* was transformed with the final insert fused by PCR using primers [103 + 104], together with the AMA1 linearized backbone.

In order to generate the strain with the translocated SphC, the fragment from the plasmid pYES2-olicP-PvSP-sphC was amplified with PCR using primers [85 + 87] and transformed into the *xyl*P*::sphG* strain (18). Transformants were streaked out on agar plates containing pyrithiamine and hygromycin, after which genomic DNA was extracted and subjected to Southern blot to confirm correct integration (Fig. S14). The probe was PCR amplified with the addition of DIG-11-UTP (Jena Bioscience, Jena, Germany) using [87 + 88] primers. To confirm the correct localization of PvHLH^SphH^-SphC, additional strains were generated—one containing the translocated SphC fused with C-terminal GFP, and second one containing only the PvHLH^SphH^ with GFP, in the background of *lcbA::DsRed*.

Generation of deletion mutants of *xyl*P::*sphG* was done by generation of separate PCR products and further assembled with three fragment PCR. *ptrA* resistance cassette was amplified from pSK275 (REF) plasmid using primers [108 + 109]. Flanks were amplified from *A. fumigatu*s gDNA with primer pairs [110 + 111] and [112 + 113] for Δ*sphB-E*, and [114 + 115] and [116 + 117] for Δ*sphA-F*. Full fragments were assembled and amplified with [110 + 113] and [114 + 117] and used for transformation of the *xyl*P::*sphG* strain. Transformants were streaked out on plates with pyrithiamine and hygromycin. Southern blot with the extracted gDNA was used to verify the mutants (Fig. S15). PCR probe was amplified using primers [114 + 118].

### Confocal microscopy

All microscopy experiments were performed on an Axio Observer Spinning Disc Confocal Microscope (Carl Zeiss, Jena, Germany) with 63×/1.2 Oil or 100×/1.4 Oil objectives. 405 nm, 488 nm, and 561 nm laser lines were used to excite the fluorescent proteins. Fungal conidia were grown overnight at 37°C in AMM as adherent cultures in ibidi well chamber slide (ibidi, Gräfelfing, Germany). Time-lapse microscopy was performed with a modified, previously described protocol (30). The culture was incubated at room temperature during the experiment, lasting for two to three hours. When necessary, fixation of fungal hyphae was achieved with a final concentration of 4% formaldehyde (AppliChem, Darmstadt, Germany). For the *A. niger,* the expression of strains was induced using up to 20 µg/mL final concentration of doxycycline.

### Automated image analysis

Confocal images recorded in the Zeiss native CZI image format were processed automatically. The images were first deconvolved, using the Huygens Professional software (v.21.10; SVI, Hilversum, the Netherlands), utilizing measured point spread functions (PSF) that were recorded from 170 nm polystyrene fluorescence beads embedded into one of the investigated samples.

The deconvolved images were saved in the Imaris .ims format (Bitplane, Zürich, Switzerland) and analyzed further using Python 3.7 libraries in the Jupyter Notebook framework, built upon a custom-designed conda environment. The applied libraries included skimage, aicsimageio, numpy, matplotlib, copy, pickle, pandas, scipy, metric_scripts, and imaris_ims_file_reader.ims.

The main steps of the colocalization workflow are summarized in Fig. S16. The two channels, red and green, were analyzed separately, due to the extra steps required by the red channel, where the thresholding had to be carried out via a multi-Otsu algorithm provided by the skimage.filters library. Here three classes were defined, instead of the two-class single Otsu (also from skimage.filters) algorithm that was applied during the green channel analysis. Representative examples of the image processing outcomes are shown in Fig. S17. Due to the noisier background of the red channel (Fig. S17E and F), its processing included an extra step of morphological opening of the binary image, followed by the multi-Otsu thresholding. The colocalization measures were calculated based on the processed images of the two channels, provided in the form of several coefficients including the Pearson’s correlation (Fig. S17, bottom nodes) (32). The output of these measures was provided for statistical analysis in the form of CSV files.

### Yeast functional complementation assay

Thermosensitive mutants *lcb1-2*, *lcb2-2*, and *tsc10-1* were transformed with the obtained circular plasmids. As a control, empty pYES2-TEF vector was additionally transformed. For the assay, an overnight culture of two transformants was prepared and shaken at 180 rpm and 30°C, except for SPT mutants where the culture was shaken at room temperature. Yeast cultures were adjusted to OD_600_ of 1 and subsequently diluted with 1:10 dilution series. About 10 µL of these dilutions were spotted onto the SD -Ura plates and incubated at 30°C, and 35°C for *tsc10-1* complementation mutants.

### Resazurin cell viability assay

Inhibition of fungal strains by myriocin was determined by measuring the fluorescence change when nonfluorescent resazurin blue dye is reduced to the fluorescent resorufin, a reaction that happens only in viable cells (37, 38). Measurements were performed in 96-well plates (BRANDplates, VWR, Darmstadt, Germany) in a CLARIOstar plate reader (BMG Labtech, Ortenberg, Germany) at 37°C, for 96 h, with measurements done every 1 h. Concentration of spores used was 10^4^ spores/mL and incubated in AMM containing either glucose or xylose and 0.002% (wt/vol) resazurin dye. Nontreated samples contained the final concentration of 1% (vol/vol) DMSO, while treated ones contained 50 µM myriocin (vol/vol, stock solution of 1 mM dissolved in DMSO). Blank did not contain any cells. Measurements were done in triplicates, with each of the samples normalized with its starting value (Fig. S8). Growth inhibition was determined by comparison of nontreated and treated samples.

### Gene expression analysis with qRT-PCR

Fungal strains were grown in AMM, containing either glucose or 2% (wt/vol) xylose shaking for 24 h on 37°C, after which the mycelium was harvested and ground. RNA was isolated using InviTrap Spin Plant RNA Mini Kit (Invitek Molecular GmbH, Berlin, Germany). Next, 1 µg of RNA was used to synthesize cDNA with the ProtoScript II first-strand cDNA synthesis kit (New England Biolabs GmbH, Frankfurt am Main Germany) with the oligo(dT) primer according to the standard protocol. For qRT-PCR, MyTaq HS Mix (Biocat, Heidelberg, Germany) was used together with 5% (vol/vol) EvaGreen Dye (Biotium, Fremont, CA) and black 96 – well plates (Applied Biosystems, Berlin, Germany). Reactions were run in a Bio-Rad CFX96 Real-Time System and analyzed with Bio-Rad CFX Maestro Software (Bio-Rad Laboratories GmbH, Feldkirchen, Germany). Expression of housekeeping genes (AFUB_013260 encoding for histone H4.1, AFUB_006770 encoding for translation elongation factor 1 alpha subunit, and AFUB_093550 encoding for actin) (55) and *sph* genes was determined using primers listed in Table S6. The annealing temperature was set to 60°C, with primer efficiencies between 90% and 110%. Relative expression was calculated with the ΔΔ*C_T_* method (56).

### SPT enzyme purification and SphA activity assay

Analysis of the SphA activity was done with previously purified FadD, SphA, and SPT enzymes and already described protocol (18, 19).

### Western blot

To analyze the cleavage pattern of the *HLH^SphH^* mutants in *S. cerevisiae* by western blot, 4 mL pre-cultures were set up in SD-Ura overnight at 30°C. On the following day, cultures were diluted to OD 0.1 in 30 mL SD-Ura and grown for 22 h. The cells were harvested and resuspended in 500 µL buffer containing 0.5 M NaCl, 0.1 M Tris, 1 mM EDTA, and 0.5 mM AEBSF, pH 8.0. Protein concentration of the samples was estimated via Bradford assay.

To detect HLH^SphH^ in *A. fumigatus* by western blot, 5 × 10^5^ spores were inoculated in 50 mL AMM and incubated for 18 h at 37°C, 180 rpm. The culture was induced with 20 µg/mL final concentration of doxycycline and further incubated for 6 h. The mycelium was filtered through Miracloth, ground in liquid nitrogen, and resuspended in 1 mL buffer containing 0.5 M NaCl, 0.1 M Tris, 1 mM EDTA, 0.5 mM AEBSF, pH 8.0. Protein concentration of the samples was estimated via Bradford assay. For immuno-detection, the GFP antibody (B-2) conjugated with HRP was hybridized (Santa Cruz Biotechnology, Dallas, USA) using iBind Flex (Thermo Scientific, Waltham, USA) for 3 h, and the membrane was imaged using Fusion FX (Vilber, Collégien, France).

### Extraction of sphingofungin intermediates and high-resolution mass spectrometry analysis

Darken pre-culture (57) was first inoculated with 5 × 10^6^ spores/mL of the respective *A. fumigatus* strains, shaking at 180 rpm and 37°C overnight. About 1 mL was used to inoculate main cultures of 20 mL V8-based medium with added 2% (wt/vol) xylose. The standing cultures were incubated at 28°C for 6 days, after which the extraction of sphingofungins intermediates was achieved by using the previously described protocol (18).

## SUPPLEMENTAL MATERIAL

Supplemental figures mbio.00195-24-s0001.pdf Figures S1-S17.

Supplemental material mbio.00195-24-s0002.docx Supplemental tables movie legends, and references.

Movie S1 mbio.00195-24-s0003.mp4 Time-lapse of SphH-GFP and LcbA-DsRed (magenta) in *A. fumigatus.*

Movie S2 mbio.00195-24-s0004.mp4 Time-lapse of SphD-GFP and LcbA-DsRed (magenta) in *A. fumigatus.*

Movie S3 mbio.00195-24-s0005.mp4 Time-lapse of SphA-GFP and LcbA-DsRed (magenta).

Movie S4 mbio.00195-24-s0006.mp4 Changes in the overlapping green volume ratio to red surfaces through time of SphH-GFP and LcbA-DsRed.

Movie S5 mbio.00195-24-s0007.mp4 3D red and green objects and cellular changes through time.

ASM does not own the copyrights to Supplemental Material that may be linked to, or accessed through, an article. The authors have granted ASM a non-exclusive, world-wide license to publish the Supplemental Material files. Please contact the corresponding author directly for reuse.
