# Supplementary material for: The spatial organization of sphingofungin biosynthesis in Aspergillus fumigatus and its cross-interaction with sphingolipid metabolism
Source: mBio. 2024 Feb 21;15(3):e00195-24. doi: 10.1128/mbio.00195-24 (PMC10936153; doi:10.1128/mbio.00195-24)
Supplement: Supplemental figures — Figures S1-S17. [file mbio.00195-24-s0001.pdf]

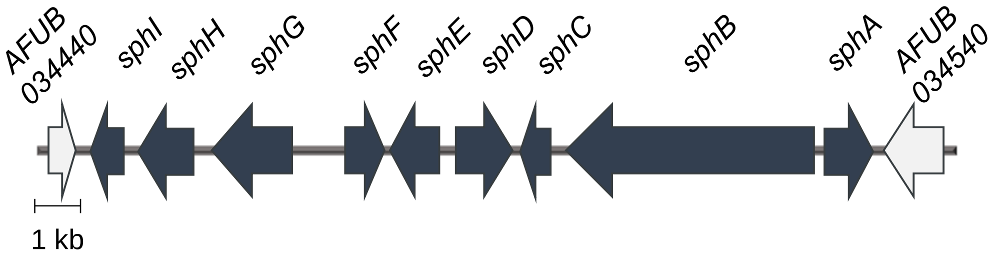

Name	Accession number	Function
-	AFUB_034540	Fungal phospholipase B-like*
sphA	AFUB_034530	Aminotransferase
sphB	AFUB_034520	Polyketide synthase
sphC	AFUB_034510	Monooxygenase
sphD	AFUB_034500	Major Facilitator Superfamily (MFS)
sphE	AFUB_034490	Acetyl transferase
sphF	AFUB_034480	Keto-reductase
sphG	AFUB_034470	$Zn(II)_2Cys_6$ transcription factor
sphH	AFUB_034460	Cytochrome P40 monooxygenase
sphI	AFUB_034450	Aldehyde reductase*
-	AFUB_034440	Shikimate 5-dehydrogenase*

*Putative function

Figure S1. Sphingofungin gene cluster in *A. fumigatus*. Shown are genes associated with the cluster and neighboring genes (white) with accession number of the genes and their function.

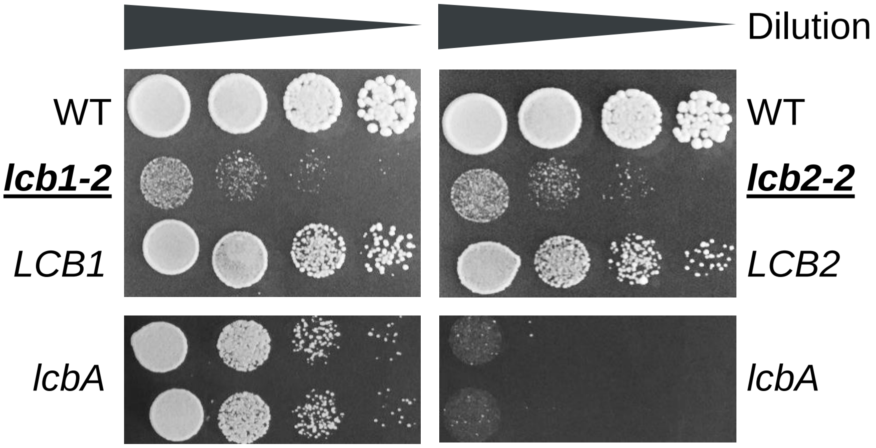

Figure S2. *S. cerevisiae* complementation assay for SPT function. LcbA from *A.fumigatus* was expressed under the control of Tef promoter in the *lcb1-2* and *lcb2-2* backgrounds. Plates were incubated on 30 °C.

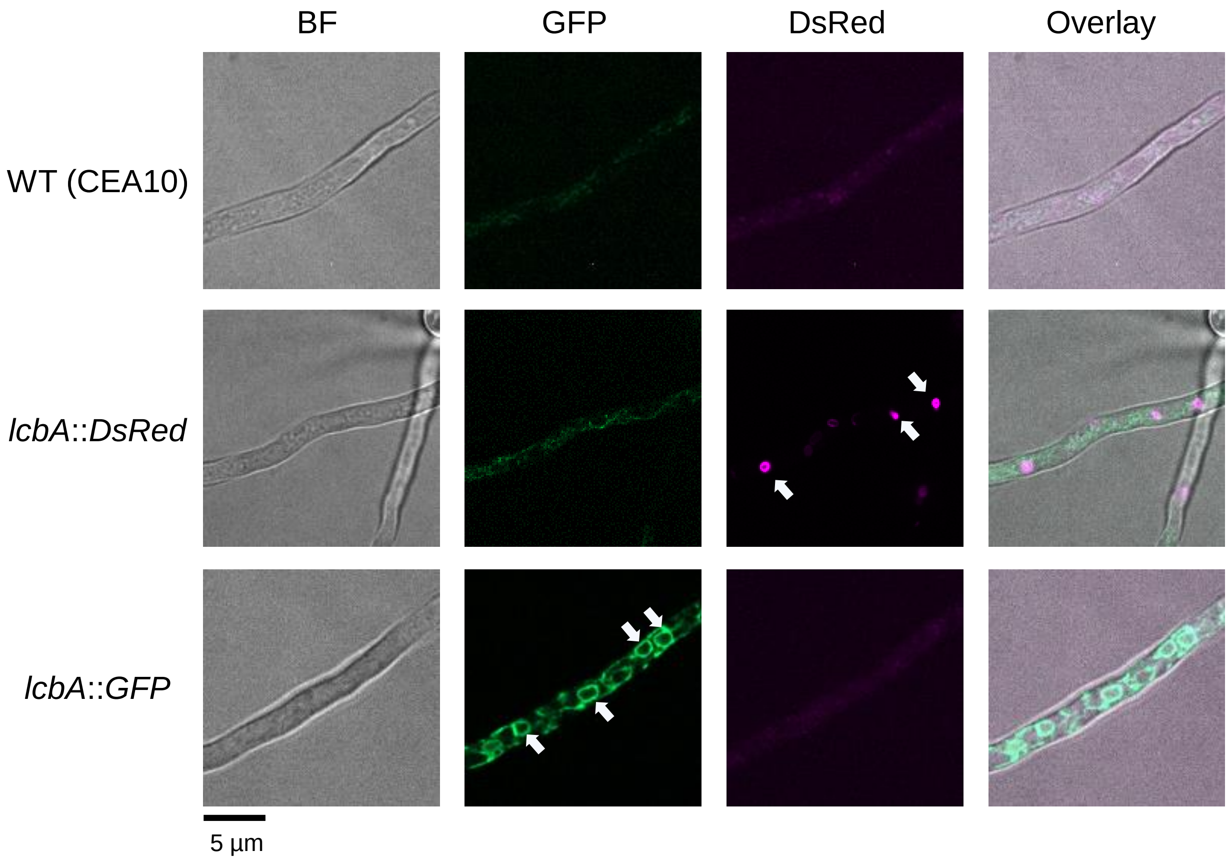

FigureS3. Control microscopy for WT and LcbA tagged with DsRed and GFP in *A. fumigatus*. Strains were analyzed for GFP and DsRed fluorescence in untreated, live cells. Individual channels and the overlay with brightfield (BF) are shown.

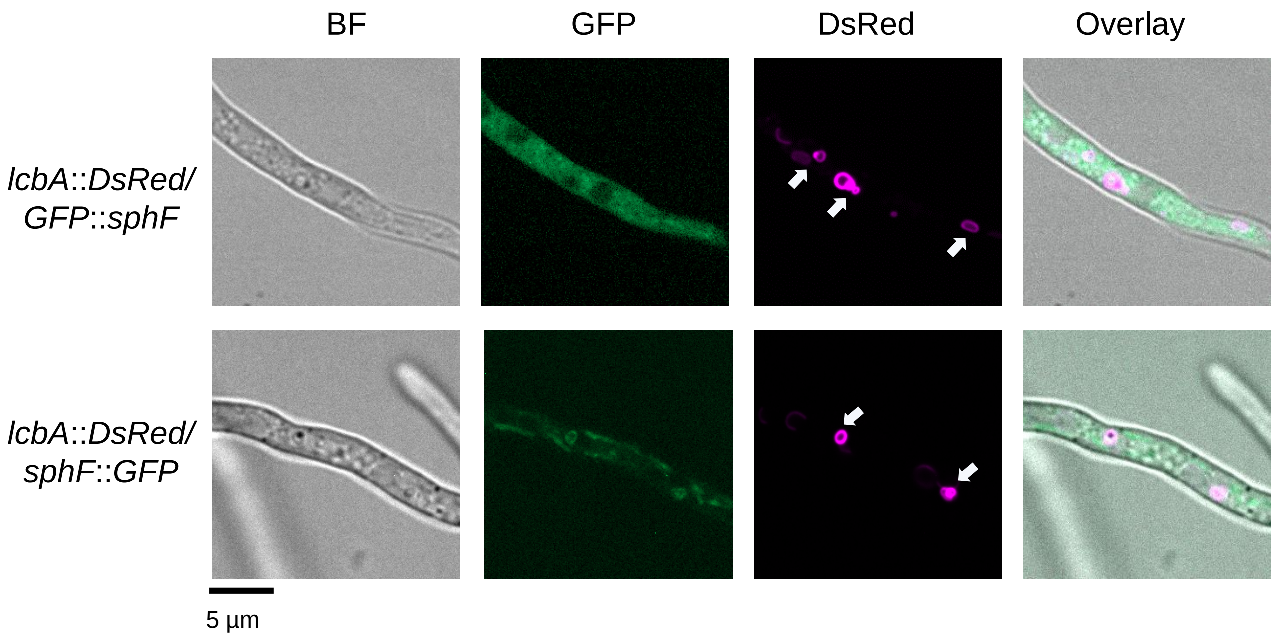

Figure S4. Tagging of whole SphF on both N- and C-terminus in *A. fumigatus*. Strains including LcbA-DsRed were analyzed for GFP and DsRed fluorescence in live cells. Shown are individual channels and the overlay with brightfield (BF).

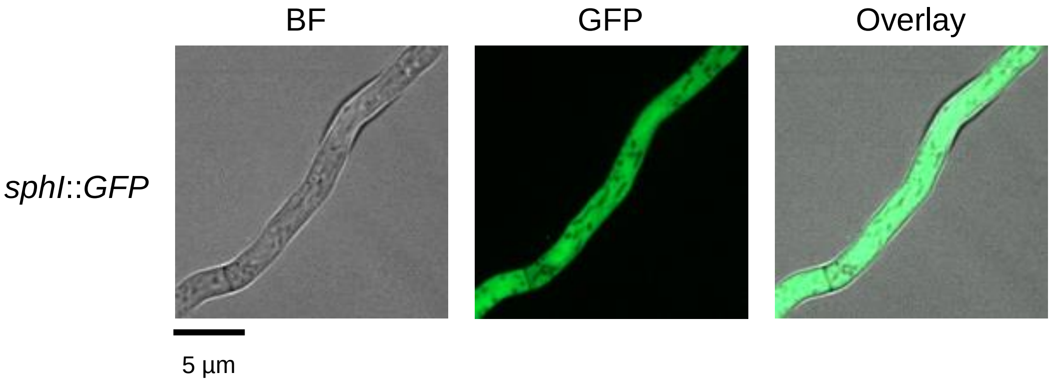

Figure S5. Localization of SphI-GFP in *A. fumigatus*. Untreated, live cells were analyzed for GFP fluorescence. Individual channels and overlay with brightfield (BF) are shown.

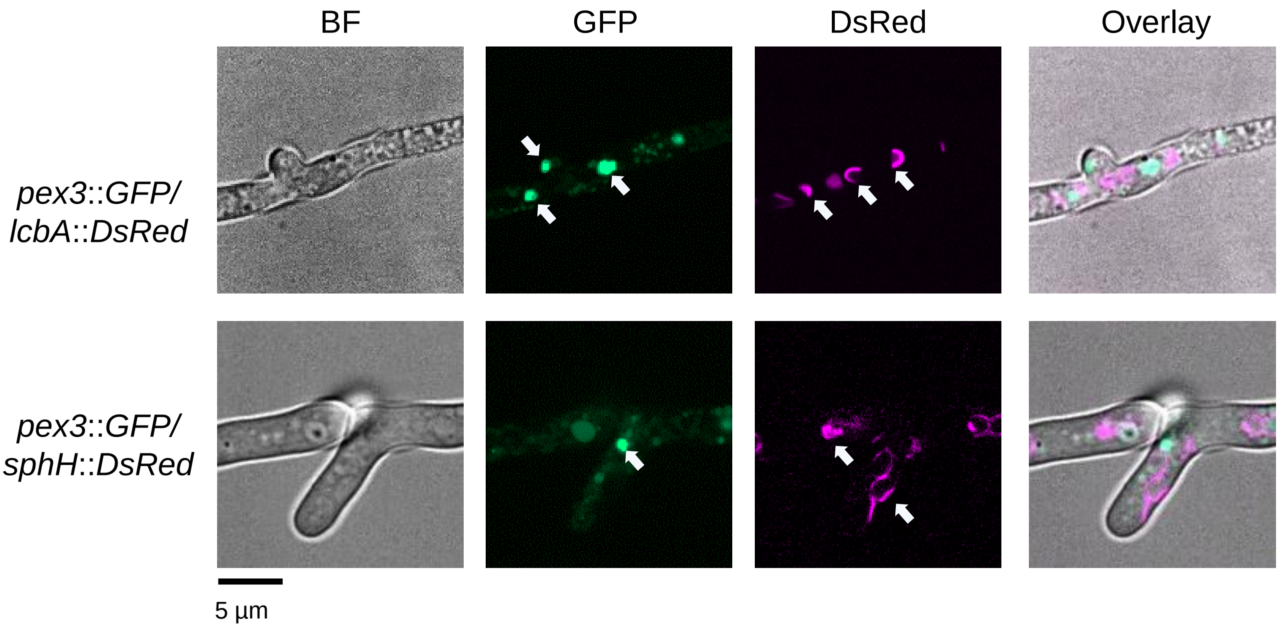

Figure S6. Co-localization of the Pex3 protein with LcbA and SphH in *A. fumigatus.* Strains were analyzed for GFP and DsRed fluorescence in live cells. Shown are individual channels and the overlay with brightfield (BF).

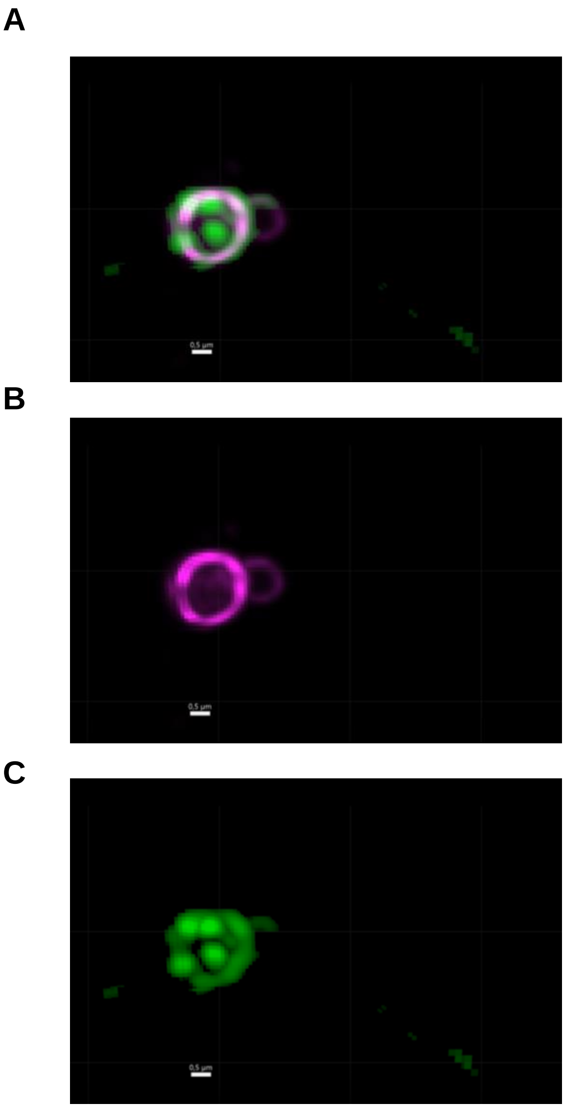

Figure S7. Colocalization of the red (LcbA) and green (SphA) channels – vesicle view. **(A)** shows the overlap of the two channels, whereas **(B)** and **(C)** presents the component images from the red and green channels, respectively.

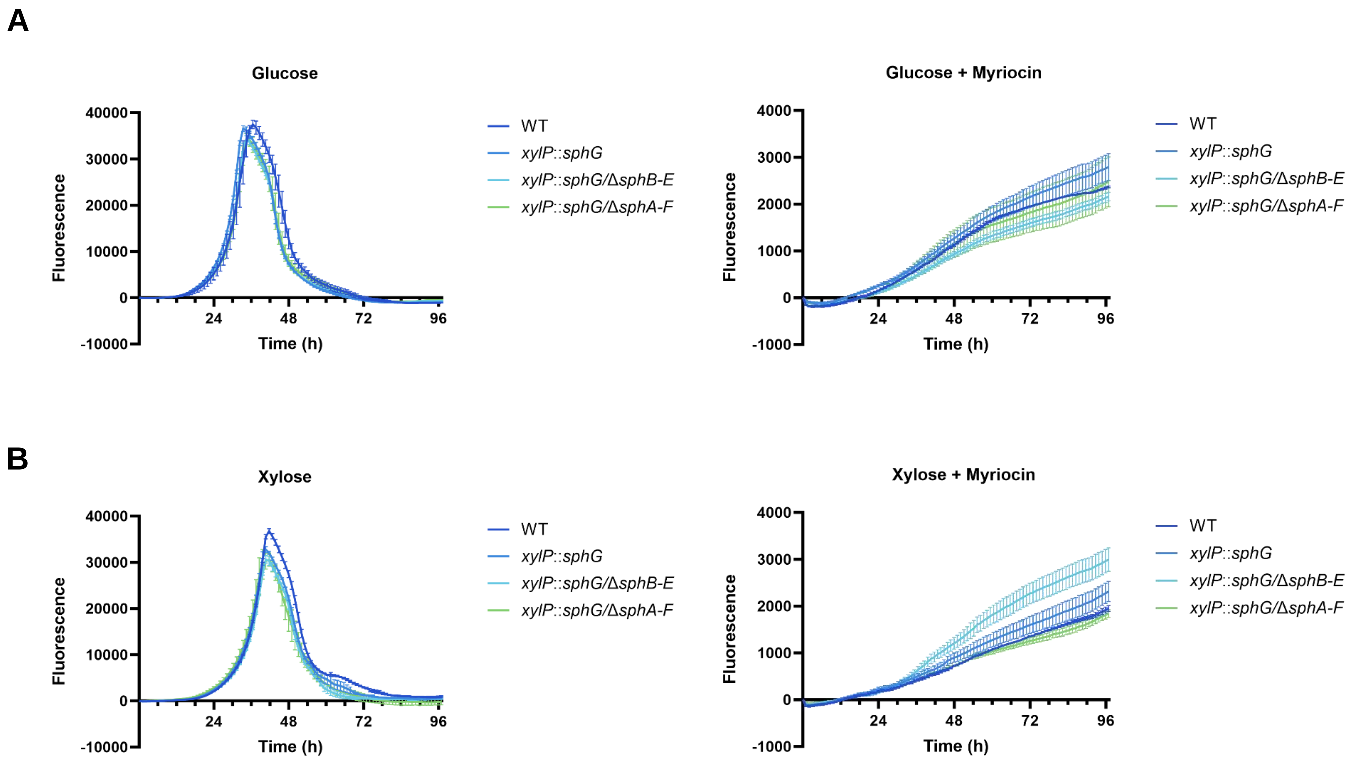

Figure S8. Resazurin cell viability assay for *A. fumigatus* strains. Shown are growth curves with treated (50 µM myriocin) and non-treated samples with the media containing either glucose (**A**) or xylose (**B**).

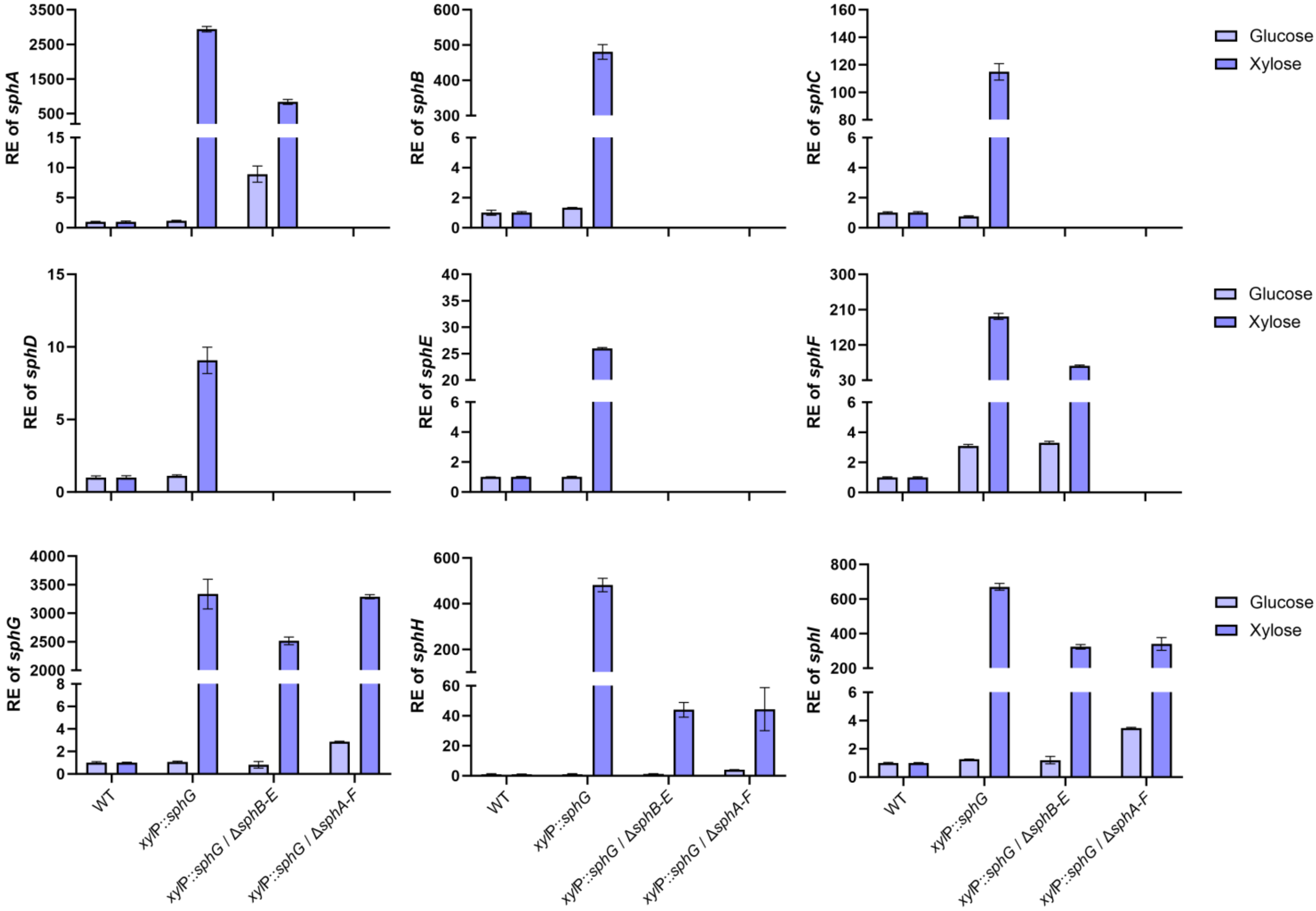

Figure S9. Gene expression levels of the *sph* cluster. The relative expression (RE) was determined by qRT-PCR after 24 hour incubation. WT gene expression was arbitrarily set to 1 for glucose and xylose – containing media. The data are means ± SEM (n = 3).

A

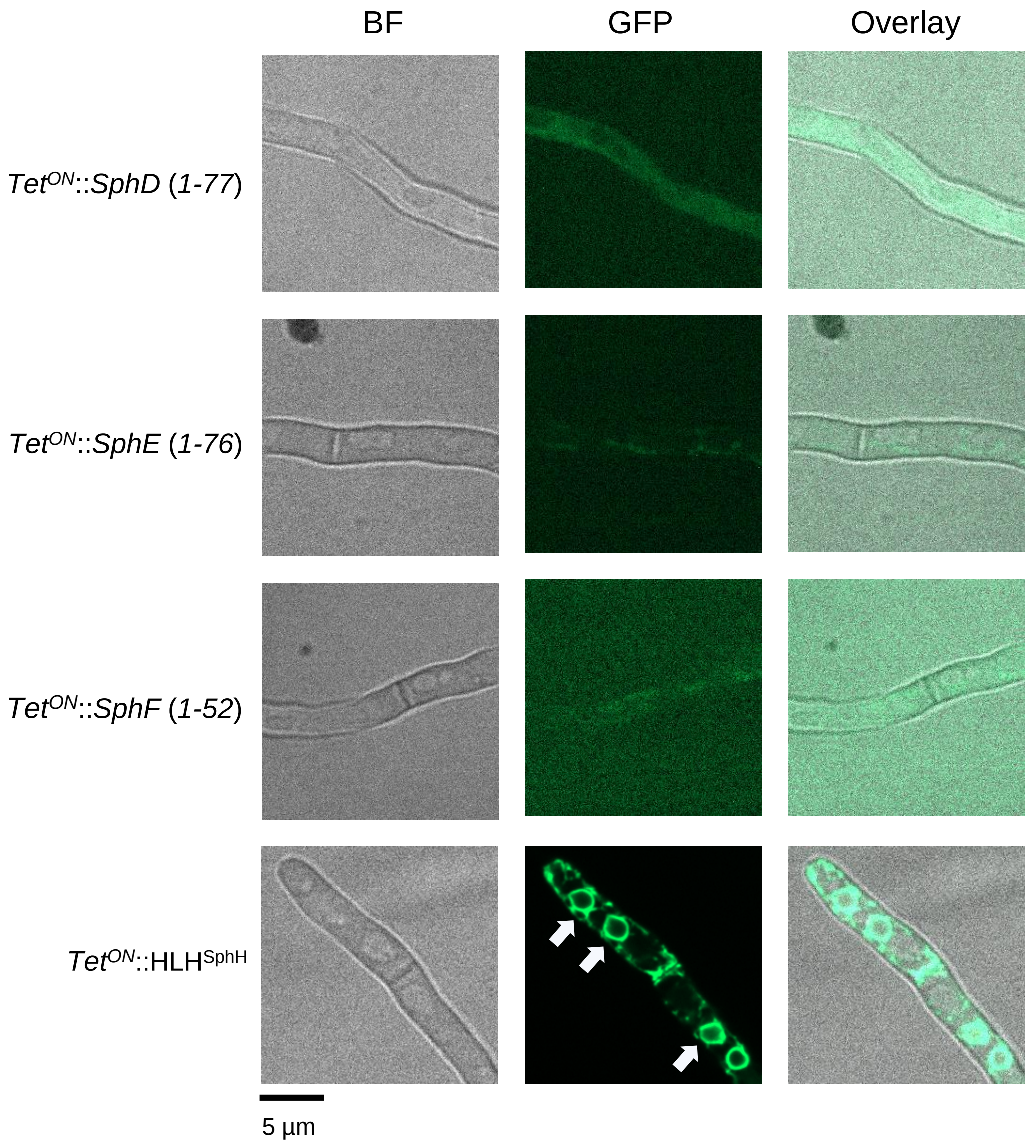

B

Source	Tested N-terminus sequence	aa
**SphD**	MGQAEFNARTDASAEISAFAVRAEPDSEPVSEKQGTAETDAETGAGGTEVPAERNGEDDVERTPKKSLSFKLAFIGL	1-77
**SphE**	MGFLRFGRESAPPPTVQSDTIIPFHYWDDDHHTRGLSFDVTFRVDDILDPEKLRCALSRLLELGDWRKLGARIRRN	1-76
**SphF**	MLERPSFPRLGAGTRHHRPLGQTGEGSDWLLAFTGISGIAGMMIPYVPWGLI	1-52
**SphH**	MGPIHNYFGVVCLGIAASVYFRPECALYGSRIATFAVLLTGIAISKLLYQLFIYPQFVTPLKHFPA	1-66
**SphH (Pv)**	MNLLGYNFGVLYIGAAAAVYFKPEYTIYDSRLLTLLAVFSIITLFKLIYNVSLYPALFTPLKEIQT	1-66

Figure S10. Microscopy of SphD, SphE, SphF, and SphH N-terminal sequences. A) Peptides were expressed in *A. niger* under control of the *Tet*^{ON} promoter. Individual channels of brightfield and GFP with an overlay are shown. **B)** Selected amino acid sequences, which were fused with GFP and tested with microscopy. Pv means the sequence is from *P. variotti*.

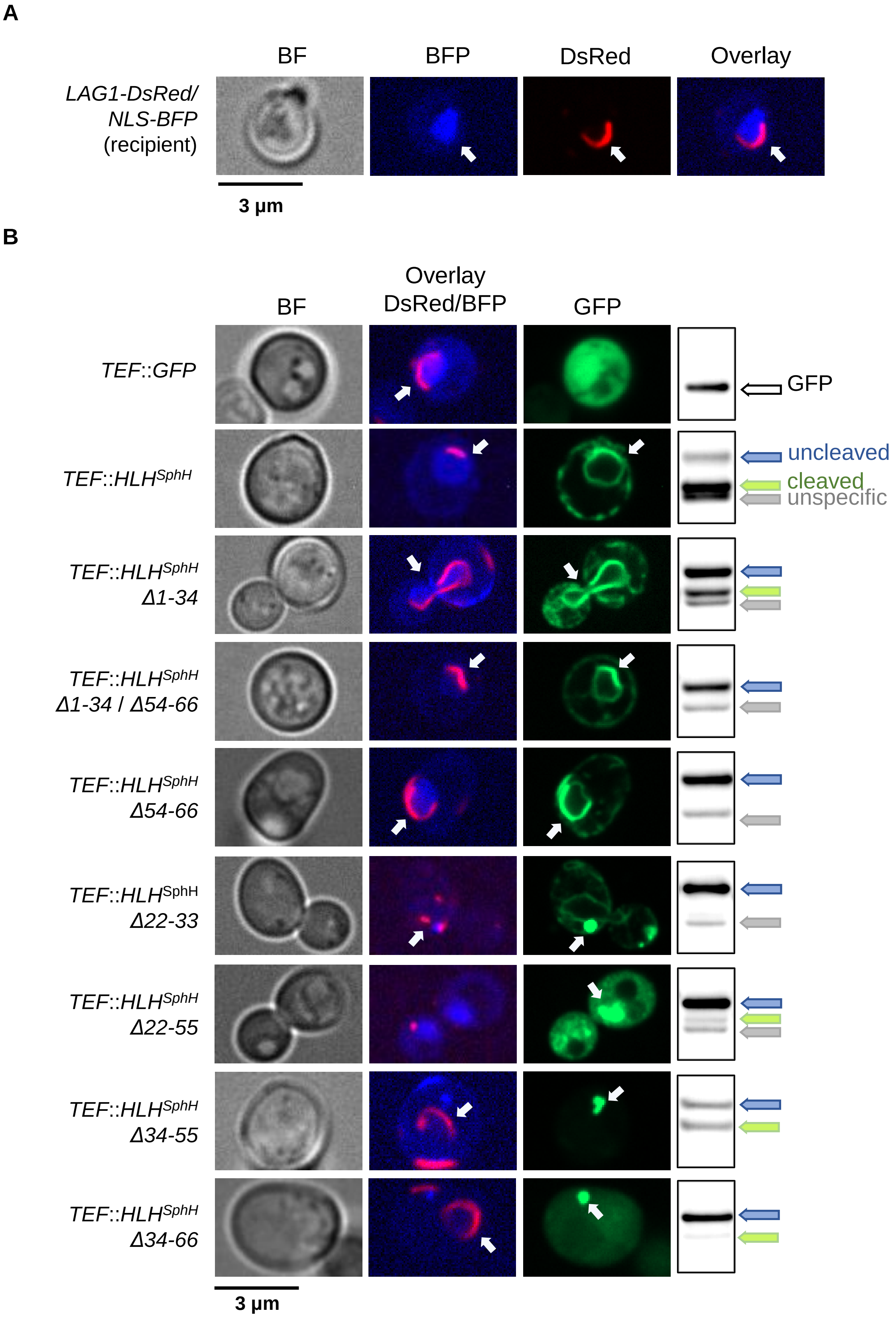

Figure S11. Confocal microscopy for the localization analysis of HLH^{SphH} mutants and Western Blot analysis of the peptides cleavage phenotype in *S. cerevisiae*. A) Localization of LAG1-DsRed in the ER and NLS-BFP in the nucleus. **B**) Localization of HLH^{SphH} mutants using a GFP fusion reporter, in the LAG1-DsRed / NLS-BFP background. (Right) Cleavage pattern for each HLH mutant is depicted by Western Blot.

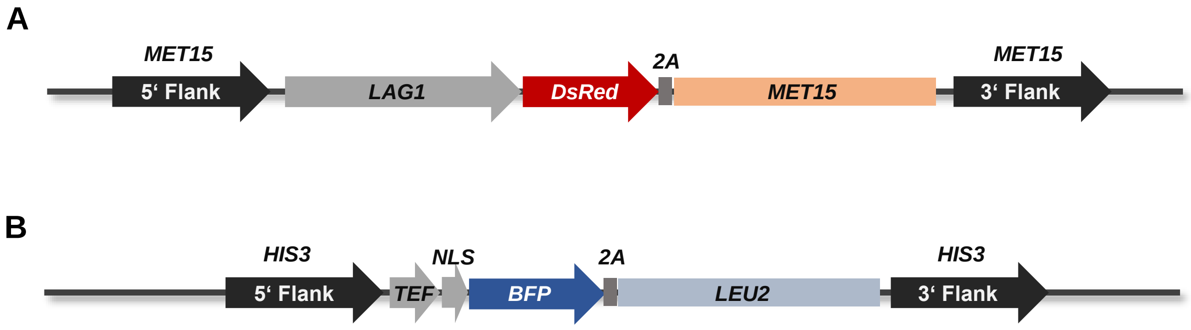

Figure S12. Strategy for *S. cerevisiae* strain with red and blue fluorescence. A) *LAG1-DsRed*::*2A*::*MET15* was integrated into the locus of the methionine (*MET15*) gene. **B)** *TEF*::*NLS-BFP*::*2A*::*LEU2* was integrated into the locus of the histidine gene (*HIS3*). **NLS**, nuclear localization signal (NLS) of the SV40 large T antigen.

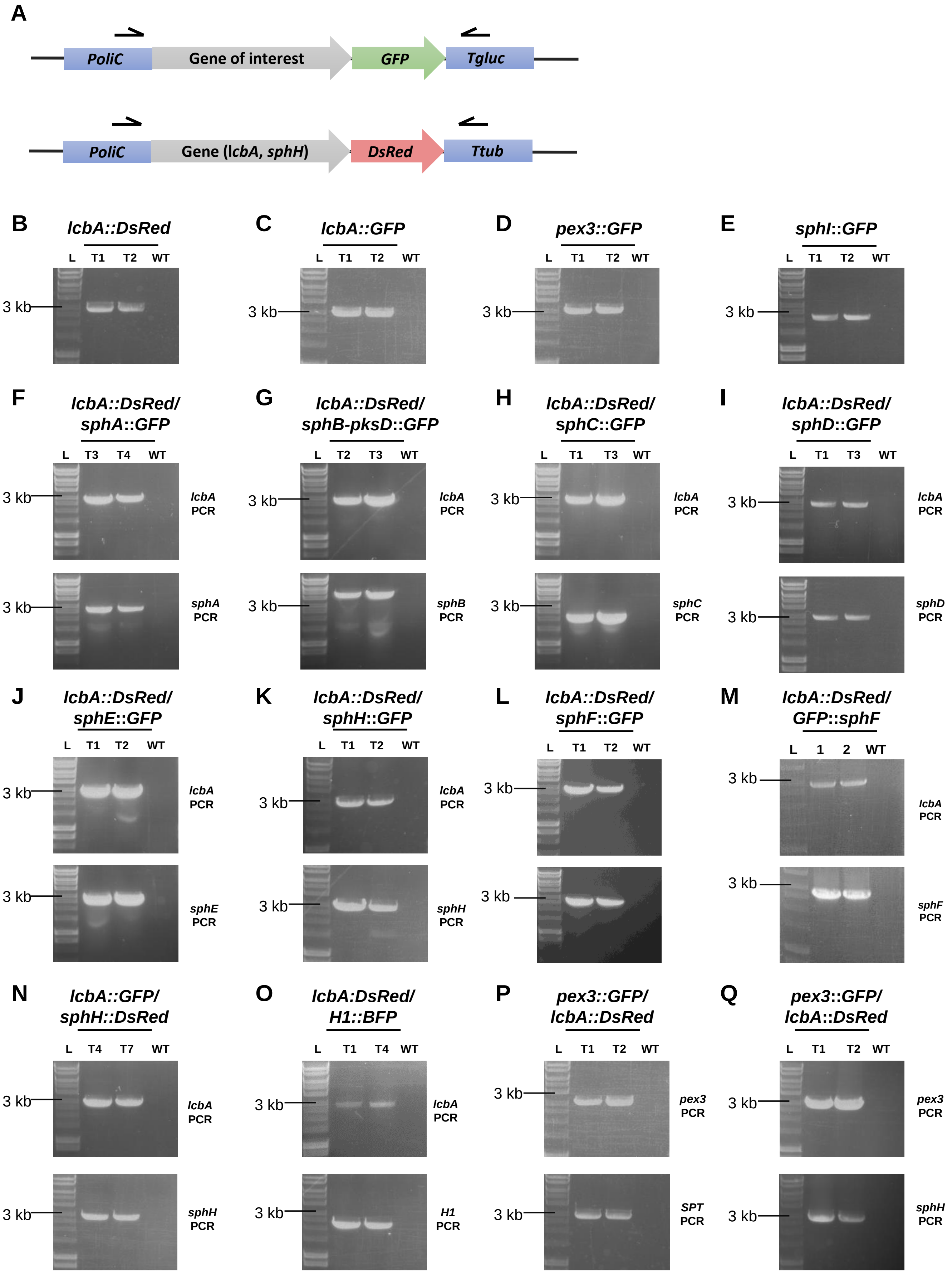

Figure S13. Verification of *A. fumigatus* strains for colocalization studies. A) Schematic representation of the part of the inserted vector with indicated control primers. **B) – Q)** Ectopic integration was verified by PCR in both single and double mutants. WT was used as a negative control and no PCR signal was detected.

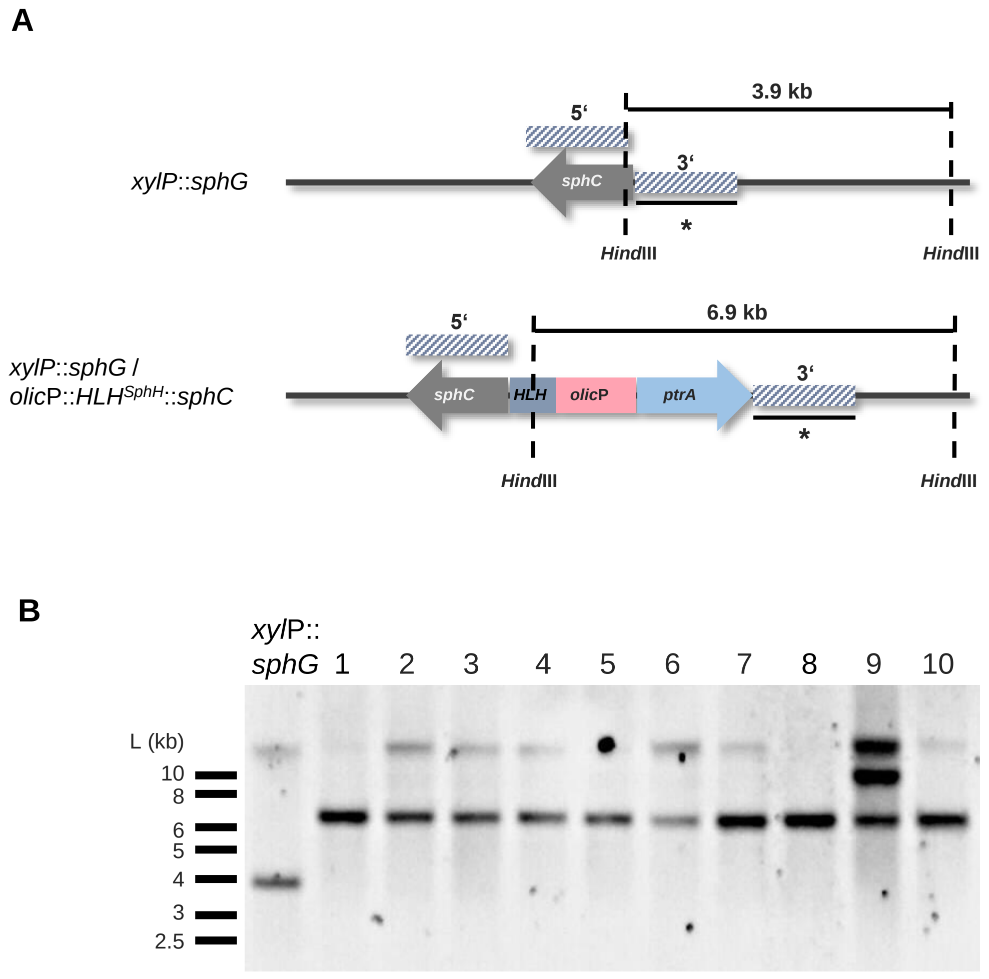

Figure S14. Verification of *A. fumigatus xyl*P::*sphG / olic*P::*HLH66*^{SphH}::*sphC* mutant. **A)** Genomic DNA was digested with *Hind*III overnight. 3’ Flank was used as a PCR – amplified probe (*). **B)** Southern Blot of *xyl*P*::sphG* and mutant strains. Expected bands for control strain at 3.9 kb and mutant strains at 6.9 kb. Black lines (**L**) indicate approximate location of Hyper Ladder 1kb on the gel.

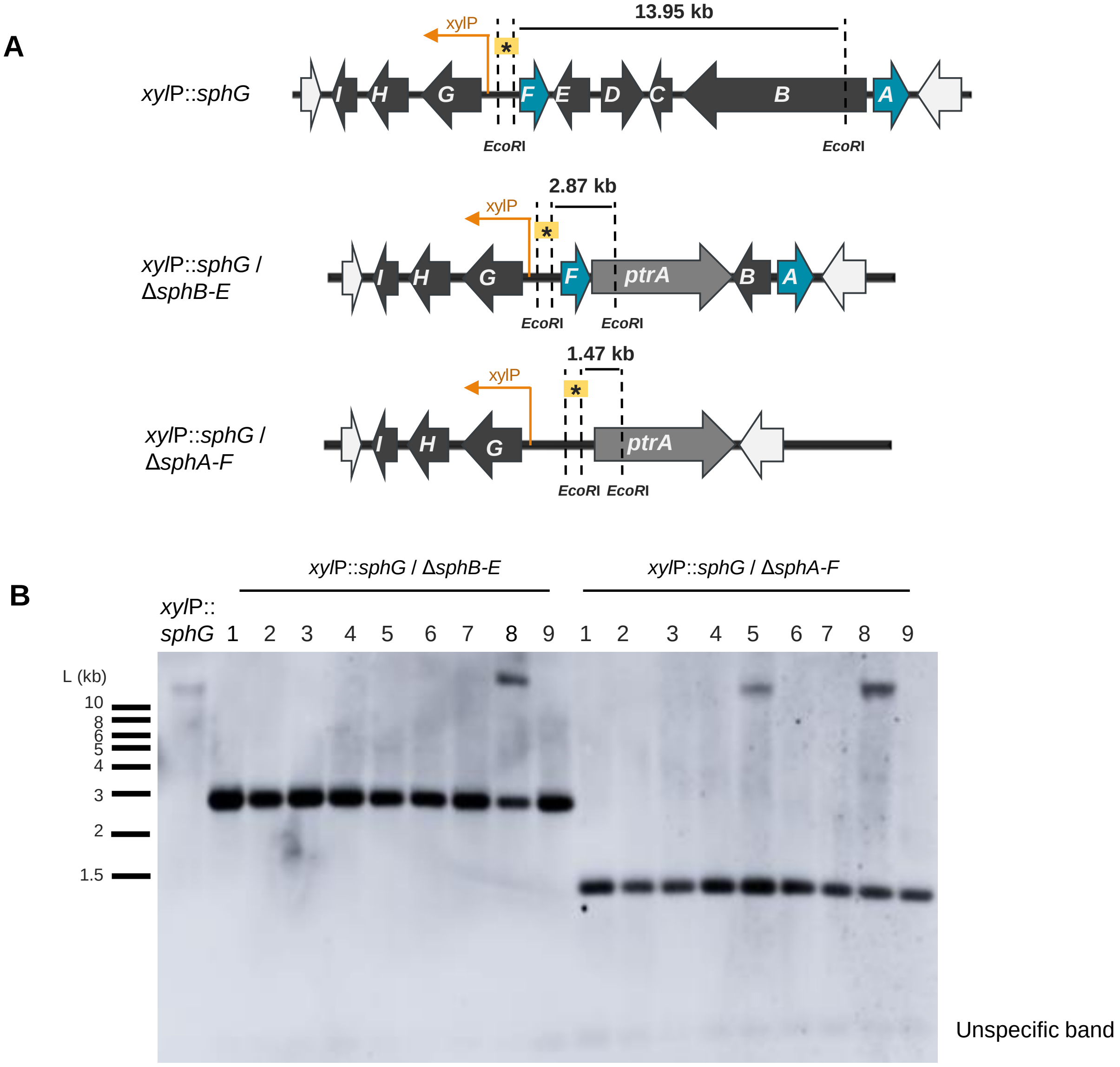

Figure S15. Verification of *xyl*P::*sphG* deletion mutants. A) Genomic DNA was digested with *Eco*RI overnight. For detection, a PCR – amplified probe was used (*). **B)** Southern Blot of *xyl*P*::sphG* and mutant strains. Expected bands for control strain at 13.95 kb, and mutant strains at 2.87 kb and 1.47 kb, respectively. Black lines (**L**) indicate approximate location of Hyper Ladder 1kb on the gel.

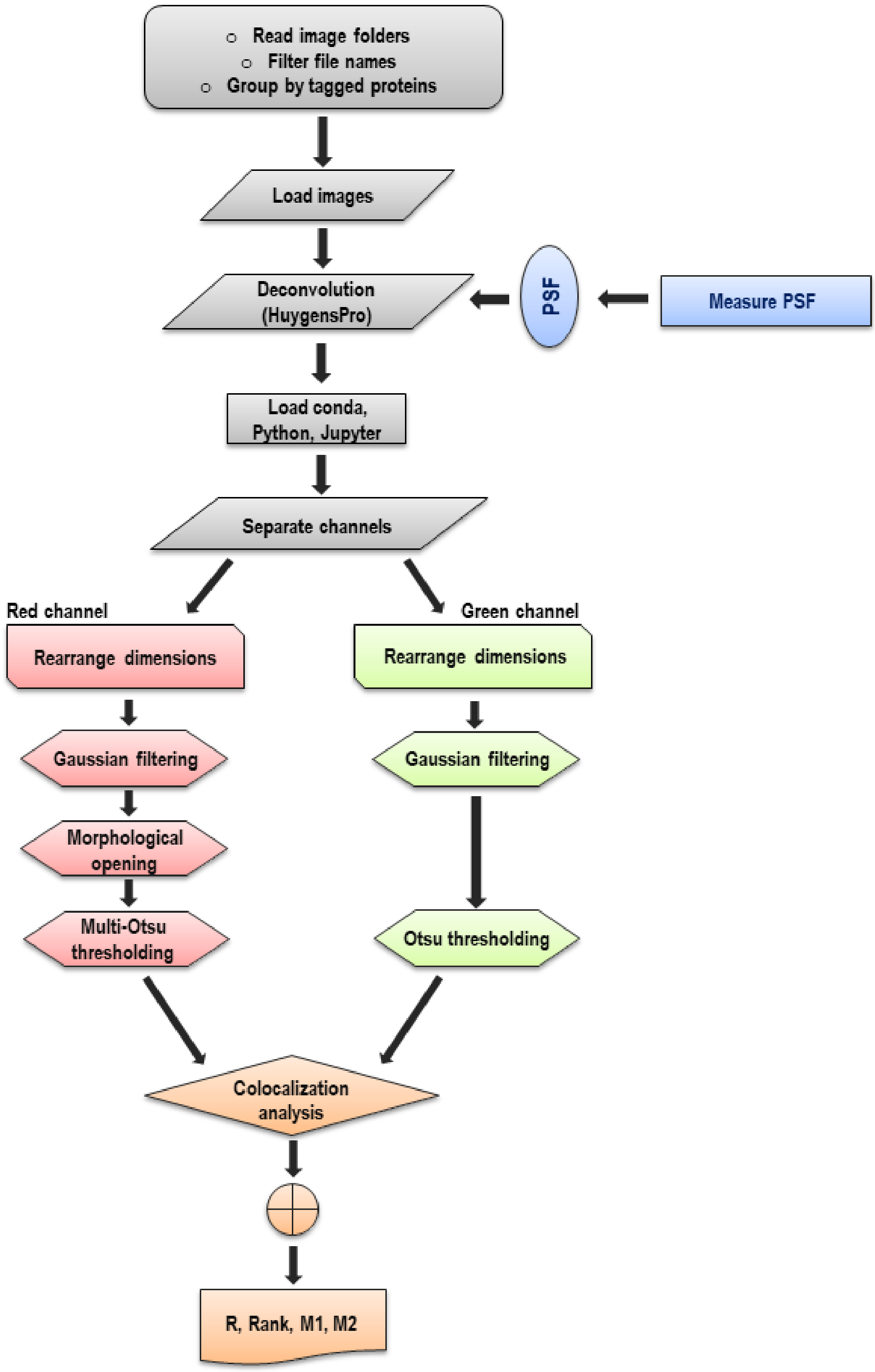

Figure S16. Flowchart of the automated image processing algorithm. The workflow processed microscopy images in the CZI format. The specifications of the applied algorithms (radii are in pixel units) were as follows: Gaussian filtering: 1.5px; segmentation algorithm: single- and multi–threshold Otsu for green (GFP) and red (DsRed) channels, respectively. For the red channel, the thresholding was preceded by a morphological dilation with a 6-pixel radius, in order to remove excessive noise.

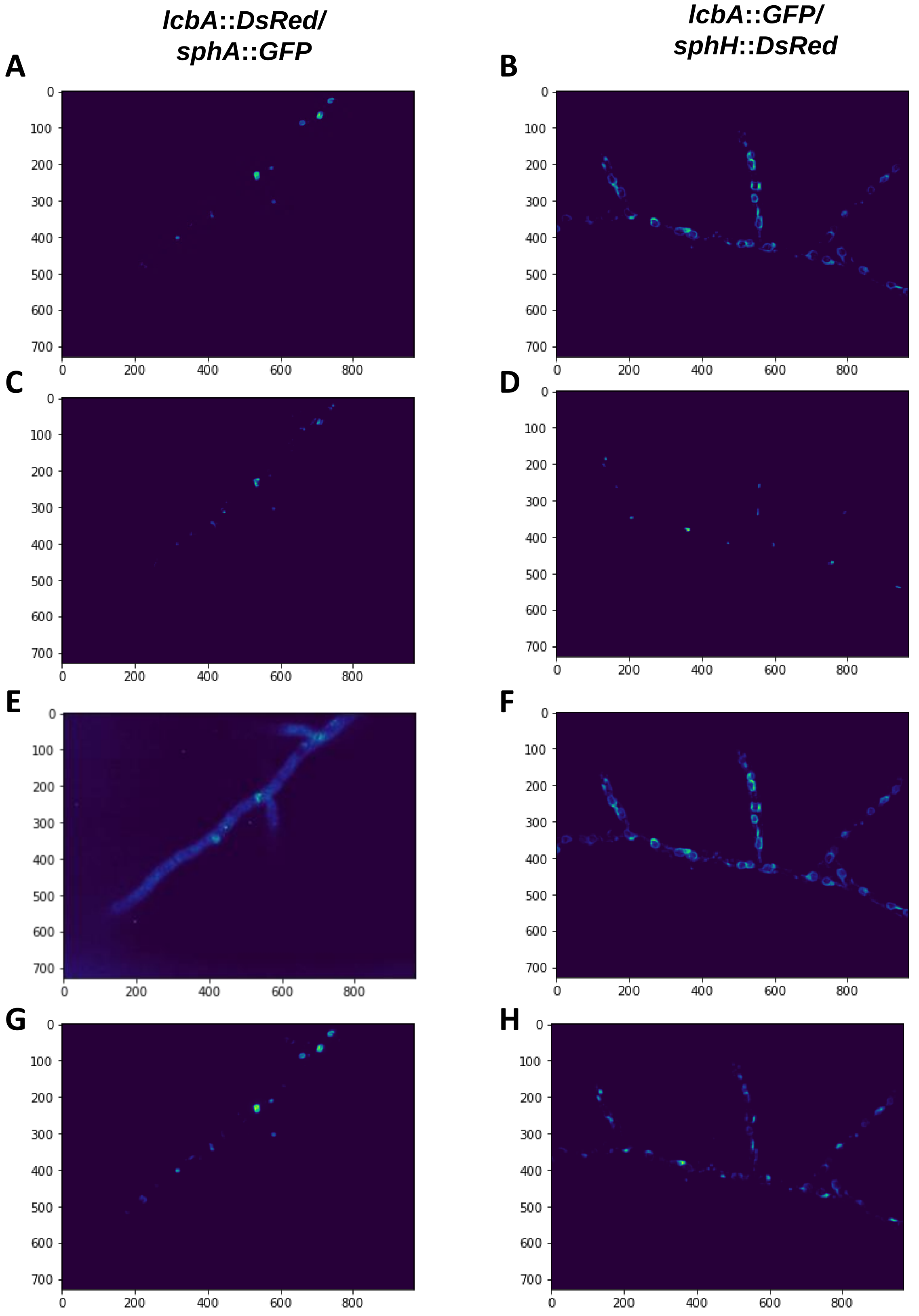

Figure S17. Representative images resulted in by the analysis workflow. (A)-(D) Representative images of the GFP (green) channel before (A,B) and after (C, D) processing. **(E)-(H)** Representative images of the red channel before (E,F) and after (G, H) processing. The processing workflow was carried out in Jupyter Notebook using Python 3.7.
