# Supplementary material for: The spatial organization of sphingofungin biosynthesis in Aspergillus fumigatus and its cross-interaction with sphingolipid metabolism
Source: mBio. 2024 Feb 21;15(3):e00195-24. doi: 10.1128/mbio.00195-24 (PMC10936153; doi:10.1128/mbio.00195-24)
Supplement: Supplemental material — Supplemental tables movie legends, and references. [file mbio.00195-24-s0002.docx]

| **Table S1. SP prediction results.** | | | | |
| --- | --- | --- | --- | --- |
| **Name** | **SignalP 6.0 (1)** | **TargetP 2.0 (2)** | **Phobius prediction** | **Microscopy** |
| *sphA* | 0.000000 | 0.000329 | 0 | Cytosol and ER-derived vesicles |
| *sphB* | 0.000001 | 0.000116 | 0 | Cytosol |
| *sphC* | 0.000000 | 0.001577 | 0 | Cytosol |
| *sphD* | 0.000031 | 0.000041 | 0 | ER-derived vesicles and  Cell-membrane |
| *sphE* | 0.000000 | 0.000162 | 0 | ER and ER-derived vesicles |
| *sphF* | 0.000922 | 0.008808 | 0 | ER-derived vesicles |
| *sphG* | 0.000000 | 0.000016 | 0 | Nucleus* |
| *sphH* | 0.000000 | 0.190720 | 0 | ER and ER-derived vesicles |
| *sphI* | 0.000118 | 0.351846 | Y | Cytosol |

*SphG was proven experimentally as a functional transcription factor (3).

| **Table S2. Fungal strains used in this study.** | | |
| --- | --- | --- |
| **Name** | **Genotype** | **Reference** |
| *A.fumigatus* **CEA10** (WT) | *MAT1-1* | (4) |
| *lcbA*::*DsRed* | CEA10; *OE*::*lcbA*::*DsRed*::ptrA | This study |
| *lcbA::DsRed* / s*phA*::*GFP* | *OE*::*lcbA*::*DsRed*::*ptrA*; *OE*::*sphA*::*GFP*::*hyg* | This study |
| *lcbA*::*DsRed* / *sphB-PksD*::*GFP* | *OE*::*lcbA*::*DsRed::ptrA; OE*::*sphB*::*GFP*::*hyg* | This study |
| *lcbA*::*DsRed* /*sphC*::*GFP* | *OE*::*lcbA*::*DsRed::ptrA; OE*::*sphC*::*GFP*::*hyg* | This study |
| *lcbA*::*DsRed* / *sphD*::*GFP* | *OE*::*lcbA*::*DsRed::ptrA; OE*::*sphD*::*GFP*::*hyg* | This study |
| *lcbA*::*DsRed* / *sphE*::*GFP* | *OE*::*lcbA*::*DsRed::ptrA; OE*::*sphE*::*GFP*::*hyg* | This study |
| *lcbA*::*DsRed* / *GFP*::*sphF* | *OE*::*lcbA*::*DsRed::ptrA; OE*::*GFP*::*sphF*::*hyg* |  |
| *lcbA*::*DsRed* / *sphF*::*GFP* | *OE*::*lcbA*::*DsRed::ptrA; OE*::*sphF*::*GFP*::*hyg* |  |
| *lcbA*::*DsRed* / *sphH*::*GFP* | *OE*::*lcbA*::*DsRed::ptrA; OE*::*sphH*::*GFP*::*hyg* | This study |
| *sphI*::*GFP* | CEA10; *OE*::*sphI*::*GFP*::*hyg* | This study |
| *lcbA*::*DsRed* / *H1*::*BFP* | *OE*::*lcbA*::*DsRed::ptrA; OE*::*h1*::*BFP*::*hyg* | This study |
| *lcbA::GFP* | CEA10; *OE*::*lcbA*::*GFP*::*hyg* | This study |
| *lcbA*::*GFP* / s*phH*::*DsRed* | *OE*::*lcbA*::*GFP*::*hyg*; *OE*::*sphH*::*DsRed::ptrA* | This study |
| *pex3*::*GFP* | CEA10; *OE*::*pex3*::*GFP*::*hyg* | This study |
| *pex3*::*GFP* / *lcbA::DsRed* | *OE*::*pex3*::*GFP*::*hyg*; *OE*::*lcbA*::*DsRed::ptrA* | This study |
| *pex3*::*GFP* / *sphH*::*DsRed* | *OE*::*pex3*::*GFP*::*hyg*;  *OE*::*sphH*::*DsRed::ptrA* | This study |
| *Af-sphA* | CEA17; *pyrG*::*TetON*::*6his*::*sphA* | (3) |
| *A. niger* **AB 4.1** | N402; *pyrG-* | (5) |
| *TetON*:: SphD (1-77) | AB 4.1; *pyrG*::*TetON*:: *SphD* (*1-77*)::*GFP* | This study |
| *TetON*:: SphE (1-76) | AB 4.1; *pyrG*::*TetON*:: *SphE* (*1-76*)::*GFP* | This study |
| *TetON*:: SphF (1-52) | AB 4.1; *pyrG*::*TetON*:: *SphF* (*1-52*)::*GFP* | This study |
| *TetON*:: *HLHSphH* | AB 4.1; *pyrG*::*TetON*::*HLHSphH*::*GFP* | This study |
| *A. fumigatus* **CEA17** Δ*akuB* | CEA17*; pyrG-,* Δ*akuB* | (6) |
| *TetON*::*GFP* | CEA17 Δ*akuB; AMA1; pyrG::TetON*::*GFP* | This study |
| *TetON*::*HLHSphH* | CEA17 Δ*akuB; AMA1; pyrG::TetON*::*HLHSphH-GFP* | This study |
| *TetON*::*HLHSphH Δ1-34* | CEA17 Δ*akuB; AMA1; pyrG::TetON*::*HLHSphH Δ1-34-*  *GFP* | This study |
| *TetON*::*HLHSphH Δ1-34* / Δ54-66 | CEA17 Δ*akuB; AMA1; pyrG::TetON*::*HLHSphH Δ1-*  *34*/Δ54-66::*GFP* | This study |
| *TetON*::*HLHSphH* Δ*54-66* | CEA17 Δ*akuB; AMA1; pyrG::TetON*::*HLHSphH* Δ*54-*  *66*::*GFP* | This study |
| *TetON*::*HLHSphH* Δ*22-33* | CEA17 Δ*akuB; AMA1; pyrG::TetON*::*HLHSphH* Δ*22-*  *33*::*GFP* | This study |
| *TetON*::*HLHSphH* Δ22-55 | CEA17 Δ*akuB; AMA1; pyrG::TetON*::*HLHSphH* Δ*22-*  *55*::*GFP* | This study |
| *TetON*::*HLHSphH* Δ*34-55* | CEA17 Δ*akuB; AMA1; pyrG::TetON*::*HLHSphH* Δ*34-*  *55*::*GFP* | This study |
| *TetON*::*HLHSphH* Δ*34-66* | CEA17 Δ*akuB; AMA1; pyrG::TetON*::*HLHSphH* Δ*34-*  *66*::*GFP* | This study |
| *TetON*:*:sphH* Δ*1-66* | *CEA17* Δ*akuB; AMA1; pyrG::TetON*::*sphH* Δ*1-66-FP* | This study |
| *TetON*::*HLHSphF* | *CEA17* Δ*akuB; pyrG*::*TetON*::*GFP*::*HLHSphF* | This study |
| ***xyl*P::*sphG*** | CEA17 Δ*akuB*; *hph*::*xyl*P::*sphG* | (3) |

| *xylP::sphG* / *olicP::PvHLH66^SphH^sphC* | *xylP*::*sphG*; *ptrA*; *olic*P::*PvHLHSphHsphC* | This study |
| --- | --- | --- |
| *lcbA*::*DsRed* / *PvHLH^SphH^sphC*::*GFP* | *OE*::*lcbA*::*DsRed*::*ptrA*; *OE*::*PvHLH^SphH^sphC*::*GFP*::*hyg* | This study |
| *lcbA*::*DsRed* / *PvSP66^SphH^*::*GFP* | *OE*::*lcbA*::*DsRed*::*ptrA*; *OE*:: *sphC*::*GFP*::*hyg* | This study |
| *xyl*P::*sphG* / Δ*sphB-E* | *xyl*P::*sphG*; Δ*sphB*; Δ*sphE*::*ptrA* | This study |
| *xyl*P::*sphG* / Δ*sphA-F* | *xyl*P::*sphG*; Δ*sphA*; Δ*sphF*::*ptrA* | This study |

| **Table S3. *S. cerevisiae* strains used in this study.** | | |
| --- | --- | --- |
| **Name** | **Genotype** | **Reference** |
| BY4741 | *MATa his3*Δ*1 leu2*Δ*0 met15*Δ*0 ura3Δ0* | (7) |
| WT | BY4741*; tef; ura3* | This study |
| *lcb1-2* | *MATa*; *ura3*; *leu2*; *his3*; *met15*; *lcb1- 2*:*kanMX* | Euroscarf |
| *lcb1-2/pYES2::TEF* | *lcb1-2; TEF; URA3* | Euroscarf |
| *lcb1-2/pTEF::LCB1* | *lcb1-2; TEF*::*LCB1; URA3* | This study |
| *lcb1-2/pTEF*::*lcbA* | *lcb1-2; TEF*::*lcbA; URA3* | This study |
| *lcb2-2* | *MATa*; *ura3*; *leu2*; *his3*; *met15*; *lcb2- 2*:*kanMX* | Euroscarf |
| *lcb2-2/pYES2*::*TEF* | *lcb1-2; TEF; URA3* | This study |
| *lcb2-2/pTEF::LCB2* | *lcb2-2*; *TEF*::*LCB2*; *URA3* | This study |
| *lcb2-2/pTEF*::*lcbA* | *lcb2-2; TEF*::*lcbA; URA3* | This study |
| *tsc10-1* | *MATa*; *ura3*; *leu2*; *his3*; *met15*; t*sc10- 1*:*kanMX* | Euroscarf |
| *tsc10-1/ pTEF*::*TSC10* | *tsc10-1; TEF*::*TSC10; URA3* | This study |
| *tsc10-1/ pTEF*::*sphF* | *tsc10-1; TEF*::*sphF; URA3* | This study |
| *TEF*::*GFP* | BY4741*; TEF*::*GFP; URA3* | This study |
| *TEF*::*HLHSphH* | BY4741*; TEF*:: *HLHSphH*::*GFP; URA3* | This study |
| *TEF*::*HLHSphH* Δ*1-34* | BY4741*; TEF*:: *HLHSphH* Δ*1-34*::*GFP; URA3* | This study |
| *TEF*::*HLHSphH* Δ*1-34* / Δ*54-66* | BY4741*; TEF*:: *HLHSphH* Δ*1-34* / Δ*54-*  *66*::*GFP; URA3* | This study |
| *TEF*::*HLHSphH* Δ*54-66* | BY4741*; TEF*:: *HLHSphH* Δ*54-66*::*GFP;*  *URA3* | This study |
| *TEF*::*HLHSphH* Δ*22-33* | BY4741*; TEF*:: *HLHSphH* Δ22*-33*::*GFP;*  *URA3* | This study |
| *TEF*::*HLHSphH* Δ*22-55* | BY4741*; TEF*:: *HLHSphH* Δ*22-55*::*GFP;*  *URA3* | This study |
| *TEF*:: *HLHSphH* Δ*34-55* | BY4741*; TEF*:: *HLHSphH* Δ*34-55*::*GFP;*  *URA3* | This study |
| *TEF*:: *HLHSphH* Δ*34-66* | BY4741*; TEF*:: *HLHSphH* Δ*34-66*::*GFP;*  *URA3* | This study |
| *LAG1*::*DsRed* | BY4741; *LAG1*::*DsRed*; *2A*::*MET15* | This study |
| *LAG1*::*DsRed* / *TEF*::*NLS*::*BFP* | *LAG1*::*DsRed*; *2A*::*MET15*; *TEF*:: *NLS*::*BFP*, *2A*::*LEU2* | This study |
| *LAG1*::*DsRed* / *TEF*::*NLS*::*BFP* / *TEF::GFP* | *LAG1*::*DsRed*; *2A*::*MET15*; *TEF*::  *NLS*::*BFP*, *2A*::*LEU2; TEF*::*GFP; URA3* | This study |
| *LAG1*::DsRed / *T*EF::*NLS*::*BFP TEF*::*HLHSphH* | *LAG1*::*DsRed*; *2A::MET15*; *TEF*::  *NLS*::*BFP; 2A*::*LEU2; TEF*::*HLHSphH; URA3* | This study |
| *LAG1*::*DsRed* / *TEF*::*NLS*::*BFP*  *TEF*::*HLHSphH* Δ*1-34* | *LAG1*::*DsRed*; *2A*::*MET15*; *TEF*:: *NLS*::*BFP; 2A*::*LEU2; TEF*::*HLHSphH* Δ*1-*  *34; URA3* | This study |
| *LAG1*::*DsRed* / *TEF*::*NLS*::*BFP*  *TEF*::*HLHSphH* Δ*1-34* / Δ*54-66* | *LAG1*::*DsRed*; *2A::MET15*; *TEF*::  *NLS*::*BFP; 2A*::*LEU2; TEF*::*HLHSphH* Δ*1- 34/*Δ*54-66; URA3* | This study |

| *LAG1*::*DsRed* / *TEF*::*NLS*::*BFP*  *TEF*::*HLHSphH* Δ*54-66* | *LAG1*::*DsRed*; *2A*::*MET15*; *TEF*::  *NLS*::*BFP; 2A*::*LEU2; TEF*::*HLHSphH* Δ*54- 66; URA3* | This study |
| --- | --- | --- |
| *LAG1*::*DsRed* / *TEF*::*NLS*::*BFP*  *TEF*::*HLHSphH* Δ*22-33* | *LAG1*::*DsRed*; *2A*::*MET15*; *TEF*:: *NLS*::*BFP; 2A*::*LEU2; TEF*::*HLHSphH* Δ22*-*  *33; URA3* | This study |
| *LAG1*::*DsRed* / *TEF*::*NLS*::*BFP*  *TEF*::*HLHSphH* Δ*22-55* | *LAG1*::*DsRed*; *2A*::*MET15*; *TEF*::  *NLS*::*BFP; 2A*::*LEU2; TEF*::*HLHSphH* Δ22*- 55; URA3* | This study |
| *LAG1*::*DsRed* / *TEF*::*NLS*::*BFP*  *TEF*::*HLHSphH* Δ*34-55* | *LAG1*::*DsRed*; *2A*::*MET15*; *TEF*:: *NLS*::*BFP; 2A*::*LEU2; TEF*::*HLHSphH* Δ*34-*  *55; URA3* | This study |
| *LAG1*::*DsRed* / *TEF*::*NLS*::*BFP*  *TEF*::*HLHSphH* Δ*34-66* | *LAG1*::*DsRed*; *2A*::*MET15*; *TEF*:: *NLS*::*BFP; 2A*::*LEU2; TEF*::*HLHSphH* Δ*34-*  *66; URA3* | This study |

| **Table S4. Plasmids used in this study.** | | |
| --- | --- | --- |
| **Name** | **Relevant features** | **Reference** |
| pNDH-OGG | *olic*P*, gfp,* T*gluc, hph* | (8) |
| pOE-sphA-GFP | pNDH-OGG, *sphA* | This study |
| pOE-sphB-PksD-GFP | pNDH-OGG, *sphB-PksD* | This study |
| pOE-sphC-GFP | pNDH-OGG, *sphC* | This study |
| pOE-sphD-GFP | pNDH-OGG, *sphD* | This study |
| pOE-sphE-GFP | pNDH-OGG, *sphE* | This study |
| pOE-sphF-GFP | pNDH-OGG, *sphF* | This study |
| pOE-GFP-sphF | pNDH-OGG, *sphF* | This study |
| pOE-sphH-GFP | pNDH-OGG, *sphH* | This study |
| pOE-sphI-GFP | pNDH-OGG, *sphI* | This study |
| pOE-SPT-GFP | pNDH-OGG, *lcbA* | This study |
| pOE-pex3-GFP | pNDH-OGG, *pex3* | This study |
| pNDH-ODT | *olic*P*, dsred,* T*tub, hph* | (8) |
| pNDP-ODT | *olic*P*, dsred,* T*tub, ptrA* | This study |
| pOE-SPT-DsRed | pNDP-ODT, *lcbA* | This study |
| pOE-sphH-DsRed | pNDP-ODT, *sphH* | This study |
| pOE-H1-BFP | *olic*P*, h1, BFÜ,* T*gluc, hph* | This study |
| pYES2-pyrG-TetON | pYES2, *pyrG*, *TetON* | (3) |
| pYES2-pyrG-TetON-GFP | pYES2-pyrG-TetON, *GFP* | This study |
| pYES2-TetON-SphD (1-77) -GFP | pYES2-pyrG-TetON-GFP, *SP77SphD* | This study |
| pYES2-TetON-SphE (1-76)-GFP | pYES2-pyrG-TetON-GFP, *SP76SphE* | This study |
| pYES2-TetON- SphF (1-52)-GFP | pYES2-pyrG-TetON-GFP, *SP52SphF* | This study |
| pYES2-TetON-HLHSphH-GFP | pYES2-pyrG-TetON-GFP, HLH*SphH* | This study |
| pYES2-TetON-GFP-HLHSphF | pYES2-pyrG-TetON-GFP, HLH*SphF* | This study |
| pYES2-TEF | *TEF, CYC1T, URA3* | (9) |
| pTEF-LCB1 | pYES2-TEF, *LCB1* | This study |
| pTEF-LCB2 | pYES2-TEF, *LCB2* | This study |
| pTEF-lcbA | pYES2-TEF, *lcbA* | This study |
| pTEF-TSC10 | pYES2-TEF, *TSC10* | This study |
| pTEF-sphF | pYES2-TEF, *sphF* | This study |
| pYES2-LAG1-DsRed / 2A-MET15 | pYES2, *LAG1*, *DsRed*, *2A*, *MET15* | This study |
| pYES2-TEF-NLS-BFP / 2A-LEU2 | pYES2, *TEF*, *NLS*, *BFP*, *2A*, *LEU2* | This study |
| pTEF-GFP | pYES2-TEF, *GFP* | This study |
| pTEF-HLHSphH-GFP | pYES2-TEF, *HLHSphH*, *GFP* | This study |
| pTEF-HLHSphH Δ1-34 | pYES2-TEF, *HLHSphH* Δ*1-34*, *GFP* | This study |
| pTEF-HLHSphH Δ1-34 / Δ54-66 | pYES2-TEF, *HLHSphH* Δ*1-34* / Δ*54-66*, *GFP* | This study |
| pTEF-HLHSphH Δ54-66 | pYES2-TEF, *HLHSphH* Δ*54-66*, *GFP* | This study |
| pTEF-HLHSphH Δ22-33 | pYES2-TEF, *HLHSphH* Δ*22-33*, *GFP* | This study |

| pTEF-HLHSphH Δ22-55 | pYES2-TEF, *HLHSphH* Δ*22-55*, *GFP* | This study |
| --- | --- | --- |
| pTEF-HLHSphH Δ34-55 | pYES2-TEF, *HLHSphH* Δ*34-55*, *GFP* | This study |
| pTEF-HLHSphH Δ34-66 | pYES2-TEF, *HLHSphH* Δ*34-66*, *GFP* | This study |
| pYES2-olicP-PvHLH^SphH^-sphC | pYES2, *ptrA*, *olic*P, *PvHLHSphH* *,sphC* | This study |
| pOE-PvHLH^SphH^-sphC-GFP | pNDH-OGG, *PvHLH^SphH^*, *sphC*, *GFP* | This study |
| pOE- PvHLH^SphH^ - GFP | pNDH-OGG, *PvHLH^SphH^*, *GFP* | This study |

| **Table S5. Primers used for cloning and fungal transformation.** | | |
| --- | --- | --- |
| **No.** | **Name** | **Sequence (5' --> 3')** |
| **1** | pOE_sphA_GFP_F | AACTCCATCACATCACAATCGATCCAAATGGCAGCTAACGGCAAGGTC |
| **2** | pOE_sphA_GFP_R | ATTACTTACCTCACCCTTGGAAACCATTGCATGCGCCGCCATCAACGC |
| **3** | pOE_sphB-PksD_GFP_F | AACTCCATCACATCACAATCGATCCAAATGACGATGGACGAAAGTACCGG |
| **4** | pOE_sphB-PksD_GFP_R | ATTACTTACCTCACCCTTGGAAACCATGAGGGTCTGGAAAGATCCTCCC |
| **5** | pOE_sphC_GFP_F | AACTCCATCACATCACAATCGATCCAAATGTGGCGCATCCCAACCGATG |
| **6** | pOE_sphC_GFP_R | ATTACTTACCTCACCCTTGGAAACCATCATCTTATCATAATCAATTG |
| **7** | pOE_sphD_GFP_F | AACTCCATCACATCACAATCGATCCAAATGGGGCAGGCCGAGTTTAAC |
| **8** | pOE_sphD_GFP_R | ATTACTTACCTCACCCTTGGAAACCATTTCCTCGAAGCGCTGGTTCCC |
| **9** | pOE_sphE_GFP_F | AACTCCATCACATCACAATCGATCCAAATGGGCTTCCTCAGATTTGGGAG |
| **10** | pOE_sphE_GFP_R | ATTACTTACCTCACCCTTGGAAACCATGGCCGCATCGTCAAGAGACATC |
| **11** | pOE_sphF_GFP_F | AACTCCATCACATCACAATCGATCCAAATGAAACCAGATCAGGACCTG |
| **12** | pOE_sphF_GFP_R | ATTACTTACCTCACCCTTGGAAACCATTTCCTCGAAGCGCTGGTTCCC |
| **13** | pOE_GFP_sphF_F | ACCTTGGGAATGGATGAACTTTACAAATTGGAGCGACCATCCTTCCCTCG |
| **14** | pOE_GFP_sphF_R | CCTAATCATACATCTTATCTACATACGTCATCGTAACCGGTTAATAGC |
| **15** | pOE_sphH_GFP_F | AACTCCATCACATCACAATCGATCCAAATGGGCCCTATTCACAATTAC |
| **16** | pOE_sphH_GFP_R | ATTACTTACCTCACCCTTGGAAACCATCCACCCTTCCACTGGAGTG |
| **17** | pOE_sphI_GFP_F | AACTCCATCACATCACAATCGATCCAAATGGCGCATCCATTGGTCTTC |
| **18** | pOE_sphI_GFP_R | ATTACTTACCTCACCCTTGGAAACCATAACGTTCGCAAACTCCAGTTGC |
| **19** | pOE_SPT_dsRed_F | AACTCCATCACATCACAATCGATCCAAATGGATATCCAGGAGACCCAACG |
| **20** | pOE_SPT_dsRed_R | ATTACTTACCTCACCCTTGGAAACCATCTTTCTCTTGCTCACAACCTTCG |
| **21** | pOE_pex3_GFP_F | AACTCCATCACATCACAATCGATCCAAATGATTGGAGCTACTAGACGCTG |
| **22** | pOE_pex3_GFP_R | ATTACTTACCTCACCCTTGGAAACCATCTCGCTCGACTCCACTGCCTTTC |
| **23** | PoliC_F | TTGATGACGTCCTCGGAGGAGGCCATTTAAATTGGATCGATTGTGATGTG |
| **24** | DsRed_SwaI_PoliC_R | TGCAGCTGTGGAGCCGCATTC |
| **25** | ptrA_PoliC_F | AATCGGGAATGCGGCTCCACAGCTGCACAATTGATTACGGGATCCC |
| **26** | ptrA_pND_R | AACCACCTCTCAGTTACTTAAAACCTCTTACTCAGCACACTCGCGCTG |
| **27** | pOE_SPT_GFP_F | AACTCCATCACATCACAATCGATCCAAATGGATATCCAGGAGACCCAAC |
| **28** | pOE_SPT_GFP_R | CTTGATGACGTCCTCGGAGGAGGCCATCTTTCTCTTGCTCACAACCTTC |
| **29** | pOE_sphH_DsRed_F | AACTCCATCACATCACAATCGATCCAAATGGGCCCTATTCACAATTAC |
| **30** | pOE_sphH_DsRed_R | CTTGATGACGTCCTCGGAGGAGGCCATCCACCCTTCCACTGGAGTG |
| **31** | pOE_H1_BFP_F | AACTCCATCACATCACAATCGATCCAAATGCCTCCCAAGAAAGCTTCCAC |
| **32** | pOE_H1_BFP_R | CGGTGAACAGCTCCTCGCCCTTGCTCACCGCCTCGCTCTTCTCAGCCTTC |
| **33** | pOE_BFP_F | GTGAGCAAGGGCGAGGAGC |
| **34** | pOE_Tgluc_BFP_R | CCTAATCATACATCTTATCTACATACGCTTGTACAGCTCGTCCATGCCG |
| **35** | pYES2_TET_R | TGTGATGTGATGGAGTTGAGATGGA |
| **36** | pYES_EcoRI_fw | AATTCTGCAGATATCCATCACACTGGCGG |
| **37** | tetON_eGFP_fw | ACCTCCATCTCAACTCCATCACATCACAGGCGCCGTTTCCAAGGGTGAGG |
| **38** | tetON_eGFP_rv | CGCCAGTGTGATGGATATCTGCAGAATTCCTAAGCGGCCGCTTTGTAAAG |
| **39** | sphD_SP77_eGFP_F | CCTCCATCTCAACTCCATCACATCACAATGGGGCAGGCCGAGTTTAACGC |
| **40** | sphD_SP77_eGFP_R | CGGGATTACTTACCTCACCCTTGGAAACGAGGCCTATAAACGCGAGTTTA |
| **41** | sphE-SP76_eGFP_F | CCTCCATCTCAACTCCATCACATCACAATGGGCTTCCTCAGATTTGGGAG |
| **42** | sphE-SP76_eGFP_F | CGGGATTACTTACCTCACCCTTGGAAACATTCCTTCGGATTCGAGCTCCG |
| **43** | sphFL-SP52_eGFP_F | CCTCCATCTCAACTCCATCACATCACAATGTTGGAGCGACCATCCTTCCC |
| **44** | sphFL-SP52_eGFP_F | CGGGATTACTTACCTCACCCTTGGAAACTATCAAGCCCCATGGAACATAT |

| **45** | sphH-SP66_eGFP_F | CCTCCATCTCAACTCCATCACATCACAATGGGCCCTATTCACAATTACTT |
| --- | --- | --- |
| **46** | sphH-SP66_eGFP_R | CGGGATTACTTACCTCACCCTTGGAAACAGCCGGAAAGTGTTTCAACGGC |
| **47** | tef_lcb1_F | CAATCTAATCTAAGTTTTAATTACAAAATGGCACACATCCCAGAGG |
| **48** | tef_lcb1_R | GTGACATAACTAATTACATGATGTTTAAACTTATTTATTAGATTCTTG |
| **49** | tef_lcb2_F | CAATCTAATCTAAGTTTTAATTACAAAATGAGTACTCCTGCAAACTATAC |
| **50** | tef_lcb2_R | GTGACATAACTAATTACATGATGTTTAAACTCAATTAACAAAATACTTG |
| **51** | tef_lcbA_F | CAATCTAATCTAAGTTTTAATTACAAAATGGATATCCAGGAGACCCAACG |
| **52** | tef_lcbA_R | TAAGCGTGACATAACTAATTACATGATCTACTTTCTCTTGCTCACAACC |
| **53** | tef_tsc10_F | CAATCTAATCTAAGTTTTAATTACAAAATGAAGTTTACGTTAGAAGACC |
| **54** | tef_tsc10_R | GTGACATAACTAATTACATGATGTTTAAACTCAGTTGGCCTTCTTGCCG |
| **55** | tef_sphF_F | CAATCTAATCTAAGTTTTAATTACAAAATGTTGGAGCGACCATCCTTCCC |
| **56** | tef_sphF_R | GTGACATAACTAATTACATGATGTTTAAACTCATCGTAACCGGTTAATAG |
| **57** | MET15.pYes.F | AATACGACTCACTATAGGGAATATTAAGCTCCACTGTAACCAGCAATGGG |
| **58** | MET15.F1.R | ATCGATAGATTTGTCCGTAGCTGATGTCATTGTATGGATGGGGGTAATAG |
| **59** | MET15.m30.F | CAGACTTCCAGCAATCTTTTG |
| **60** | MET15.pYes.R | CATAACTAATTACATGATGCGGCCCTCTAGCACGCTGCTTTGGAACCAAG |
| **61** | 2A.F.21 | GGCTCTGGCGCCACCAACTTC |
| **62** | met15_R | TCATGGTTTTTGGCCAGCG |
| **63** | Lag1_F | ATGACATCAGCTACGGACAA |
| **64** | dsRED.2A.R | GAGGAGAGAGAAGTTGGTGGCGCCAGAGCCAGCGGCCGCCAGGAACAGG |
| **65** | His3.F1 | GCTGTAATACGACTCACTATAGGGAATATTCGCTGTTGGTGCCGTAGAC |
| **66** | His3.F2 | AAGAGTAAAAAAGGAGTAGAAACATTTTGACTTTGCCTTCGTTTATCTTG |
| **67** | His3.F3 | GCCGAAGAAGTTAAGAAAATCCTTGCTTAATGACACCGATTATTTAAAGC |
| **68** | His3.F4 | CATAACTAATTACATGATGCGGCCCTCTAGCCACTGGAATATCATCGTAG |
| **69** | PyesTef2_Tef2-R | TTTGTAATTAAAACTTAGATTAGAT |
| **70** | HIS3.tCYC1-R | CATAACTAATTACATGATGCGGCCCTCTAGCTACATAAGAACACCTTTGG |
| **71** | SV40.tef.F | TAGCAATCTAATCTAAGTTTTAATTACAAAATGGACAAGGCCGAGCTGAT |
| **72** | SV40.BFP.R | CCCGGTGAACAGCTCCTCGCCCTTGCTCACGCCGGAGCCGCCGGCGGTGC |
| **73** | LEU2.End.R | TTAAGCAAGGATTTTCTTAACTTC |
| **74** | eBFP.F | GTGAGCAAGGGCGAGGAGCTG |
| **75** | eBFP.2A.R | GAGGAGAGAGAAGTTGGTGGCGCCAGAGCCCTTGTACAGCTCGTCCATGC |
| **76** | Met_6His_Rv | GTGATGGTGATGGTGATGCAT |
| **77** | SP66_6His-F35_Fw | TTCAATGCATCACCATCACCATCACTTTGCCGTTCTGTTGACAGG |
| **78** | SP66_I53-pJET_Fw | AAAGCTGCTCTATCAGTTATTCATCGGAAATATGGTTTCCAAGGG |
| **79** | SP66_I53_Rv | GATGAATAACTGATAGAGCAGCTTT |
| **80** | SP66_F21-T34_Fw | CGGCATAGCCGCAAGTGTCTACTTCACCTTTGCCGTTCTGTTGAC |
| **81** | SP66_F21_Rv | GAAGTAGACACTTGCGGCTATGCC |
| **82** | SP66_A33-Q56_Fw | CGCCCTCTACGGCTCCAGAATCGCGCAATTCGTCACGCCGTTGAA |
| **83** | SP66_A33_Rv | CGCGATTCTGGAGCCGTAGA |
| **84** | SP66_A33-pJET_Fw | CCCTCTACGGCTCCAGAATCGCGGGAAATATGGTTTCCAAGGGTG |
| **85** | BsSphH_SP_SphC_5F | GTAATACGACTCACTATAGGGAATATTTTAGAACACATCATTCAGGAGC |
| **86** | BsSphH_SP_SphC_5R | TTCACCCCACTAAAAGAGATACAAACGTGGCGCATCCCAACCGATGTCCC |
| **87** | BsSphH_SP_SphC_3F | TGTGTAACAAGAAAGATGCAAGAGGCCTTGGAGTACTGCAGGAAGGTTCC |
| **88** | BsSphH_SP_SphC_3R | GACATAACTAATTACATGATGCGGCCCGACCTACTTGAGCAGTGCCAAAG |
| **89** | BsSphH_SP_F | CGTTTGTATCTCTTTTAGTGGG |
| **90** | BsSphH_SP_R | ATGAATCTTTTAGGGTATAATTTTG |

| **91** | BS_PoliC_F | ACCAAAATTATACCCTAAAAGATTCATTTGGATCGATTGTGATGTGATGG |
| --- | --- | --- |
| **92** | BS_PoliC_R | TGGGATCCCGTAATCAATTGGCCTGAGTGCAGCTGTGGAGCCGCATTCCC |
| **93** | pSK275_ptrAterm_fw | GGCCTCTTGCATCTTTCTTG |
| **94** | pSK275_ptrAprom_rev | CTCAGGCCAATTGATTACGG |
| **95** | PvSphH_GFP_F | AACTCCATCACATCACAATCGATCCAAATGAATCTTTTAGGGTATAATTT |
| **96** | PvSphH_GFP_R | ATTACTTACCTCACCCTTGGAAACCATCATCTTATCATAATCAATTGTAT |
| **97** | Pv_GFP_R | ATTACTTACCTCACCCTTGGAAACCATCGTTTGTATCTCTTTTAGTGGGG |
| **98** | poliC_Seq_F2 | GGGAGACGTATTTAGGTGCTAGGG |
| **99** | tgluc_Seq_R2 | CCGCCCTCTTTTGTCTTCCGC |
| **100** | ttub_Seq_R2 | GGGTATCGACGATGTGTAGTCG |
| **101** | TetON_SwaI1-6His- SP66_Fw | TCAACTCCATCACATCACAATTTCAATGCATCACCATCACCATCAC |
| **102** | SwaI(2)_eGFP_Rv | CCTCACCCTTGGAAACCATATTTCC |
| **103** | eGFP-AMA_pal1_Rv | CTCCAGTCATCTGCGGAACATATACTGGGCCCTTAAGCGGCCGCTTTGTAAAGTT |
| **104** | AMA_pal2-pyrG_Fw | CCAGTCATCTGCGGAACATATACTGGGCCCGCTGCACCTTCAGACACTCC |
| **105** | pYes2_cyc1T_fw | ATCATGTAATTAGTTATGTCACG |
| **106** | eGFP-SphF_SP70_Fw | CTTGGGAATGGATGAACTTTACAAAGTTGCGCGGGTCGACCGAGG |
| **107** | SphF_SP_C_term_R | CGTGACATAACTAATTACATGATCTATCGTAACCGGTTAATAGCATCTTC |
| **108** | ptrA_F | TCTTTCTTGTTACACATAATTATTC |
| **109** | ptrA_R | CAATTGATTACGGGATCCCATTG |
| **110** | sphF_GFP_5diag | GTCACAATATTCGCACGACG |
| **111** | Dsph.B-E.F1.R | TTCGTTACCAATGGGATCCCGTAATCAATTGGTCTCTTGACGATGCGGCC |
| **112** | Dsph.B-E.F2.F | TTCTAGAATAATTATGTGTAACAAGAAAGACCATGGCGGTCTCCGCGTAG |
| **113** | sphB-Rev | ATGACGATGGACGAAAGTAC |
| **114** | AFUB_034480-P1 | CGAATATAGCTTGCGAGAGC |
| **115** | Dsph.A-F.F1.R | TCGTTACCAATGGGATCCCGTAATCAATTGGTTCGGTGTAAACGGGAAGG |
| **116** | Dsph.A-F.F2.F | TTCTAGAATAATTATGTGTAACAAGAAAGACCGCGCAGGAGATTTACGCG |
| **117** | sphA_GFP_3diag | GGATGACATCGCCGTCTAC |
| **118** | sphF5Flank_sphFc_rev | ACGAGGGAAGGATGGTCGCTCCAACATCGTCCATCAAATAAGGTCTAGTC |
|  |  |  |
|  |  |  |

| **Table S6. Primers used for qRT-PCR.** | | |
| --- | --- | --- |
| **Name** | **Sequence (5' --> 3')** | **Target gene** |
| AfH4.1_qRT_F | CTCGCTCGTCGTGGTGGTGTC | Histone H4.1 |
| AfH4.1_qRT_R | GCCTTGTCTCTTCAGAGCGTAGACG |  |
| AfEF1_qRT_F | GTCGTTTCGCCGTCCGTGAC | Transcription elongation factor alpha subunit 1 |
| AfEF1_qRT_R | CGGCCTTGGTGACCTTACCG |  |
| AfAct1_qRT_F | CCCAGAGCTCCAGCTTGGAGAAG | Actin |
| AfAct1_qRT_R | CCAGACCCAGAACGCTAGGCTG |  |
| sphA_qRT_F | CGCCGTGGTCCCTATTGACTATC | *sphA* |
| sphA_qRT_R | GATCTTGCCCTTCTCGCCACC |  |
| sphB_qRT_F | CACAACAATTCTACGGTGGCGGC | *sphB* |
| sphB_qRT_R | GTCGCTTCCCGATCTCGACCG |  |
| sphC_qRT_F | CGGCGGAAGCACATCCGTTC | *sphC* |
| sphC_qRT_R | CATGCTGCAAATCCTCCATCGC |  |
| sphD_qRT_F | CTCGTCGGCCTCACAATCTCTGC | *sphD* |
| sphD_qRT_R | GACCGGAACGTCGATTGACCG |  |
| sphE_qRT_F | GGACAATGCTCATCATCCAGCCC | *sphE* |
| sphE_qRT_R | CAGTTCCTTGGATGCAGGACGG |  |
| sphF_qRT_F | CGAGGATTCATTGGCTGCGAGTC | *sphF* |
| sphF_qRT_R | CCTCCTCAACACCGGCACCAC |  |
| sphG_qRT_F | CGCCCATGAGTTCCATCACCTC | *sphG* |
| sphG_qRT_R | GACTCGCCCAGGGCTTCCATAG |  |
| sphH_qRT_F | GCACGCAGGTCGTCATTTCG | *sphH* |
| sphH_qRT_R | GCTAGTTCGGCCTTGGCGAAG |  |
| sphI_qRT_F | GTGGCGGATGTCATTCAGCG | *sphI* |
| sphI_qRT_R | GACCACATCCCGCACCATCTG |  |
|  |  |  |

# Supplementary movies.

**Movie S1. Time-lapse of SphH-GFP and LcbA-DsRed (magenta) in *A.fumigatus*.** Shown are the two channels merged. Dynamic movement of vesicles is noticeable. As the hyphal branch forms, the vesicles move towards the tip.

**Movie S2. Time-lapse of SphD-GFP and LcbA-DsRed (magenta) in *A.fumigatus*.** Shown are merged channels. The transporter, SphD is prominent in the plasma membrane along with further locations – ER, vesicles with LcbA present and smaller, mobile vesicles.

**Movie S3. Time-lapse of SphA-GFP and LcbA-DsRed (magenta).** The video is zoomed in to a specific vesicle and area where the enzymes colocalize.

**Movie S4. Changes in the overlapping green volume ratio to red surfaces through time of SphH-GFP and LcbA-DsRed.** Five-dimensional representation of the overlapping volume ratio between the green (SphH-GFP) and red (LcbA-DsRed) fluorescence channels. The 3-D objects in the video represent the outside surfaces of the segmented bright zones of green fluorescence, with their color corresponding to the overlap ratio between the green and red channels. The color scale bar at the bottom right corner of the video represents the range of overlap between 0.00 and 0.68. The size of the objects denotes the overlapping volume between the two channels. The time scale of the video corresponds to the running time of the image acquisition series. The X, Y, Z axis of the 3-D coordinate system of the video depict the time index of the acquisition, the mean fluorescence intensity of the green channel (channel 2), and the mean fluorescence intensity of the red channel (channel 5), respectively. The color of the 3-D tracks changes from blue to red as the timepoint of the recording progresses from zero to 180 minutes.

**Movie S5. 3D red and green objects and cellular changes through time.** Green represents SphH masked over the red LcbA objects.

**References:**

1. Teufel F, Almagro Armenteros JJ, Johansen AR, Gislason MH, Pihl SI, Tsirigos KD, Winther O, Brunak S, von Heijne G, Nielsen H. 2022. SignalP 6.0 predicts all five types of signal peptides using protein language models. Nat Biotechnol 40:1023-1025.
2. Almagro Armenteros JJ, Salvatore M, Emanuelsson O, Winther O, von Heijne G, Elofsson A, Nielsen H. 2019. Detecting sequence signals in targeting peptides using deep learning. Life Sci Alliance 2.
3. Bissell AU, Rautschek J, Hoefgen S, Raguž L, Mattern DJ, Saeed N, Janevska S, Jojić K, Huang Y, Kufs JE, Herboeck B, Guo H, Hillmann F, Beemelmanns C, Valiante V. 2022. Biosynthesis of the sphingolipid inhibitors sphingofungins in filamentous fungi requires aminomalonate as a metabolic precursor. ACS Chem Biol 17:386-394.
4. Fedorova ND, Khaldi N, Joardar VS, Maiti R, Amedeo P, Anderson MJ, Crabtree J, Silva JC, Badger JH, Albarraq A, Angiuoli S, Bussey H, Bowyer P, Cotty PJ, Dyer PS, Egan A, Galens K, Fraser-Liggett CM, Haas BJ, Inman JM, Kent R, Lemieux S, Malavazi I, Orvis J, Roemer T, Ronning CM, Sundaram JP, Sutton G, Turner G, Venter JC, White OR, Whitty BR, Youngman P, Wolfe KH, Goldman GH, Wortman JR, Jiang B, Denning DW, Nierman WC. 2008. Genomic islands in the pathogenic filamentous fungus *Aspergillus fumigatus*. PLoS Genet 4:e1000046.
5. van Hartingsveldt W, Mattern IE, van Zeijl CM, Pouwels PH, van den Hondel CA. 1987. Development of a homologous transformation system for *Aspergillus niger* based on the *pyrG* gene. Mol Gen Genet 206:71-75.
6. da Silva Ferreira ME, Kress MR, Savoldi M, Goldman MH, Hartl A, Heinekamp T, Brakhage AA, Goldman GH. 2006. The akuB(KU80) mutant deficient for nonhomologous end joining is a powerful tool for analyzing pathogenicity in Aspergillus fumigatus. Eukaryot Cell 5:207-211.
7. Brachmann CB, Davies A, Cost GJ, Caputo E, Li J, Hieter P, Boeke JD. 1998. Designer deletion strains derived from *Saccharomyces cerevisiae* S288C: a useful set of strains and plasmids for PCR-mediated gene disruption and other applications. Yeast 14:115- 132.
8. Schumacher J. 2012. Tools for *Botrytis cinerea*: New expression vectors make the gray mold fungus more accessible to cell biology approaches. Fungal Genet Biol 49:483- 497.
9. Hoefgen S, Lin J, Fricke J, Stroe MC, Mattern DJ, Kufs JE, Hortschansky P, Brakhage AA, Hoffmeister D, Valiante V. 2018. Facile assembly and fluorescence-based screening method for heterologous expression of biosynthetic pathways in fungi. Metab Eng 48:44-51.
